# Interactive phenotyping of large-scale histology imaging data with HistomicsML

**DOI:** 10.1038/s41598-017-15092-3

**Published:** 2017-11-06

**Authors:** Michael Nalisnik, Mohamed Amgad, Sanghoon Lee, Sameer H. Halani, Jose Enrique Velazquez Vega, Daniel J. Brat, David A. Gutman, Lee A. D. Cooper

**Affiliations:** 1 Department of Biomedical Informatics, Emory University School of Medicine, Atlanta, USA; 2 Department of Neurology, Emory University School of Medicine, Atlanta, USA; 3 Emory University School of Medicine, Atlanta, USA; 4 Department of Pathology & Laboratory Medicine, Emory University School of Medicine, Atlanta, USA; 5 Winship Cancer Institute, Emory University, Atlanta, USA; 6 Department of Biomedical Engineering, Georgia Institute of Technology/Emory University School of Medicine, Atlanta, GA USA

## Abstract

Whole-slide imaging of histologic sections captures tissue microenvironments and cytologic details in expansive high-resolution images. These images can be mined to extract quantitative features that describe tissues, yielding measurements for hundreds of millions of histologic objects. A central challenge in utilizing this data is enabling investigators to train and evaluate classification rules for identifying objects related to processes like angiogenesis or immune response. In this paper we describe HistomicsML, an interactive machine-learning system for digital pathology imaging datasets. This framework uses active learning to direct user feedback, making classifier training efficient and scalable in datasets containing 10^8^+ histologic objects. We demonstrate how this system can be used to phenotype microvascular structures in gliomas to predict survival, and to explore the molecular pathways associated with these phenotypes. Our approach enables researchers to unlock phenotypic information from digital pathology datasets to investigate prognostic image biomarkers and genotype-phenotype associations.

## Introduction

Slide scanning microscopes can digitize entire histologic sections at 20X–40X objective magnification, generating expansive high-resolution images containing 10^9^+ pixels. For cancer tissues, these images contain important biologic and prognostic information, capturing the diverse cytologic elements involved in angiogenesis, immune response, and tumor/stroma interactions. Image analysis algorithms can mine whole-slide images to delineate objects like cell nuclei, and to extract 10s–100s of quantitative features that describe the shape, color, and texture of each object. These histology-omic or “histomic” features can be used to train machine-learning algorithms to classify important elements like tumor-infiltrating lymphocytes, vascular endothelial cells, or fibroblasts. Identifying these elements in tissues requires considerable expertise, and imparting this knowledge to algorithms enables precise characterization of large imaging datasets in ways not possible by subjective visual assessment. Quantitative measures of the abundance, morphologies and spatial patterns of these elements can help investigators understand relationships between histologic phenotypes and survival, treatment response, and underlying molecular mechanisms. Studies that generate whole slide images can yield histomic features for 10^8^+ objects, and a central challenge in utilizing this data is in enabling domain experts to train classification rules and to evaluate their accuracy. With each image containing up to 10^6^+ discrete objects, facilitating interaction with domain experts requires fluid navigation of gigapixel images, visualization of derived image segmentation boundaries, mechanisms to intelligently acquire training data from experts, and to visualize classifications for millions of objects.

Histopathology image analysis has received significant attention with algorithms having been developed to predict metastasis^1^, survival^2–6^, grade^7,8^, and histologic classification^9–11^, and to link histologic patterns with genetic alterations or molecular disease subtypes^12–14^. Many algorithms demonstrate scientific or potential clinical utility, but few directly engage domain experts in analyzing histomic data^15,16^. Inputs are typically acquired offline by presenting a small collection of manually selected image sub regions to an expert for labeling or annotation. Tools like ImageJ^17^ and CellProfiler^18,19^ provide interactive image analysis and machine learning capabilities for high content screening and traditional microscopy images with limited fields of view, but are not equipped to handle whole-slide images or the massive amounts of image analysis metadata that can be extracted from these images. Enabling experts to directly interact with machine learning algorithms on large datasets creates a feedback loop that has been shown to improve prediction accuracy and user experience in general applications^20–24^. In this feedback paradigm, the expert iteratively improves a classification rule by correcting or confirming predictions on unlabeled examples, cycling between labeling and training and prediction. Active learning extends this paradigm by identifying and labeling the examples that provide the most benefit to the classifier in each cycle. This approach seeks to increase the diversity of labeled examples used for classifier training, and avoids labeling redundant examples that are unlikely to improve performance. The challenge in utilizing active learning with histomic data is in building software with the scalable visualization and machine-learning capabilities described above.

We previously developed a basic software prototype to establish the feasibility of active learning classification with whole-slide imaging datasets^25^. This prototype developed important technology for visualizing whole-slide images and image analysis metadata via the web, but lacked critical features needed for dissemination as a tool and was not extensively validated. In this paper we describe the histomics machine-learning system (HistomicsML), a deployable software system that builds on this prototype to provide key features for training accurate histologic classifiers: (i) A deployable Docker software container that avoid complex software installation procedures (https://hub.docker.com/r/histomicsml/active/) (ii) New tools for creating, sharing and reviewing labeled data and ground-truth validation sets, and for validating classifiers (iii) A web-based interface that fluidly displays gigapixel images containing 10^6^+ image analysis objects (iv) Active-learning algorithms for improved training efficiency and accuracy. HistomicsML is an open-source project (https://github.com/CancerDataScience/HistomicsML). Using imaging, clinical and genomic data from The Cancer Genome Atlas (TCGA), we validate this system by demonstrating development of an accurate classifier of vascular endothelial cell nuclei with minimal training data. We use this classifier to describe the phenotypes of microvascular structures in gliomas, and show that these phenotypes predict survival independent of both grade and molecular subtype. Finally, we identify molecular pathways associated with disease progression through integrated pathway analysis of mRNA expression data.

## Results

### Active learning classification software for histology imaging datasets

**Figure 1:**
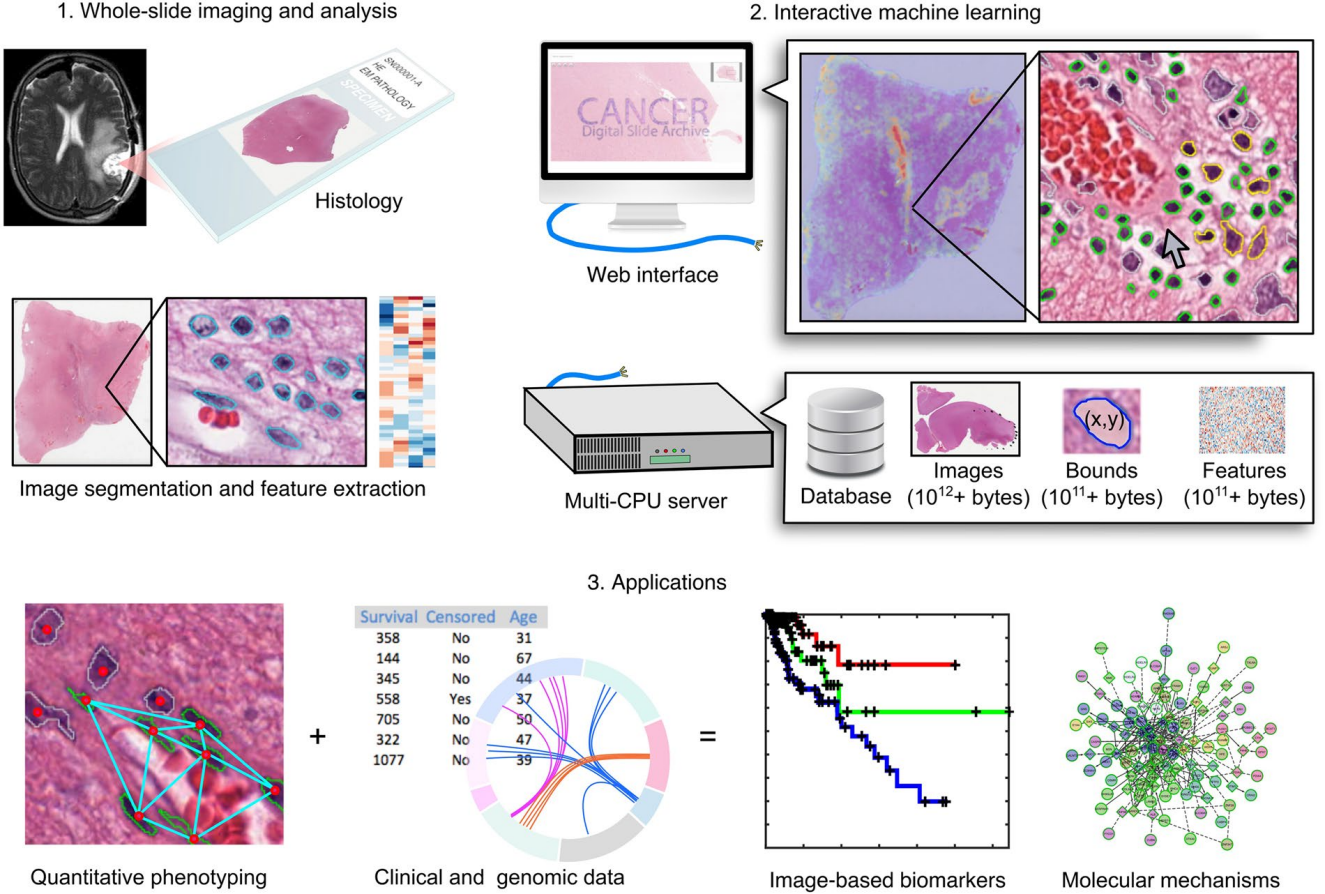
An interactive machine-learning framework for phenotyping histology images. Digitized whole-slide images of tissue sections can be analyzed to extract features describing the shape, texture and staining characteristics of histologic structures like cell nuclei. We created a software framework that enables experts to identify important histologic elements like tumor infiltrating lymphocytes or vascular endothelial cells in these images through interactive training of machine learning classifiers. A browser-based interface provides point-and-click interaction with datasets containing 10^8^+ objects for training classification rules. A multi-CPU server manages the images and boundary and feature data and provides the computational power for visualization and analysis. Classifications generated with this framework can be used to describe the phenotypes associated with cancer-related processes like angiogenesis and lymphocytic infiltration, and to investigate phenotype-genotype associations and phenotypic prognostic biomarkers.

An overview of the software system is presented in Fig. 1. Image segmentation algorithms are used to delineate histologic objects like cell nuclei in whole-slide images, and a histomic feature profile is extracted to describe the shape, texture, and staining characteristics of each delineated object (see Figure S1). Images, features, and object boundaries are stored in a database and disk array to support visualization and machine-learning analysis. A web-browser interface enables users to rapidly train classification rules and review their predictions in large datasets containing 10^8^+ objects. A multiresolution image viewer provides zooming and panning of gigapixel images and dynamically displays object boundaries. A caching and pre-fetching strategy is used to display boundaries for objects in the current field of view and to fluidly handle panning events (see Figure S2). Boundaries are color-coded to indicate their predicted class (e.g. green - endothelial cell nuclei) or membership in the training set (e.g. yellow – labeled). Users can refine the classification rule by clicking objects in the viewport to label and add them to the set of training examples. Screen captures of the interfaces are provided in Figure S3.

**Figure 2:**
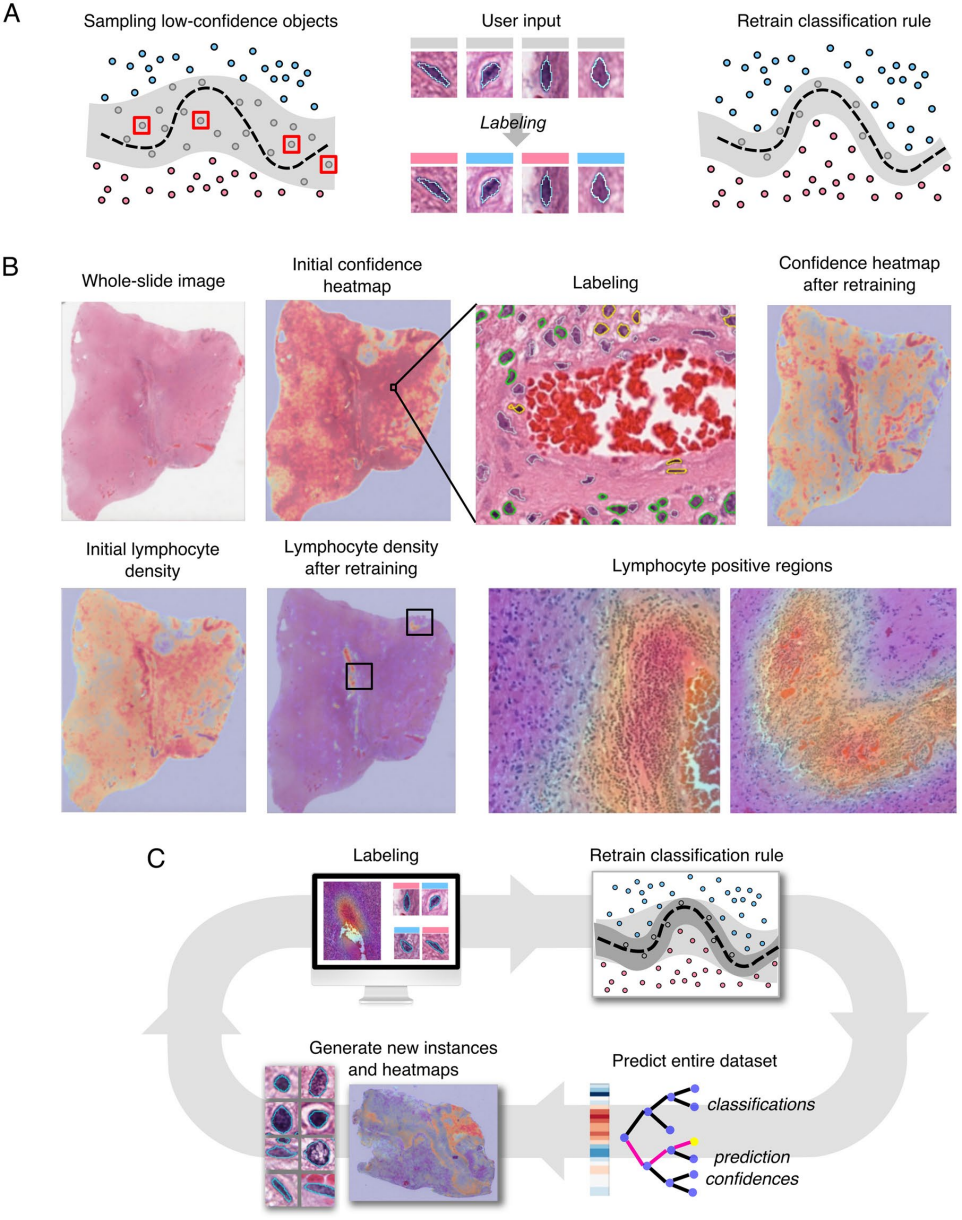
Active learning for efficient classification rule training. (**A**) (Left) A classification rule aims to learn an unknown decision boundary (black) that separates classes of objects in feature space. A margin (gray) surrounding this boundary contains objects with low prediction confidence that are difficult for the rule to classify. (Center) Instance-based learning presents unlabeled low-confidence objects to the user for labeling. (Right) Retraining the classification rule with these labels shrinks the margin towards the decision boundary improving classification accuracy. (**B**) Heatmap-based learning directs users to image regions that are enriched with low confidence objects for labeling. (Top) Correcting prediction errors (yellow) in low-confidence regions (red) and retraining reduces the number of low-confidence objects. (Bottom) Classification rule specificity is improved by re-training. Here the heatmaps indicate the density of cells positively classified as lymphocytes before and after retraining. (**C**) Active learning is an iterative process: the user first labels objects guided by active learning, then the classification rule is retrained and applied to the entire dataset, and lastly new instances and heatmaps are generated.

The active-learning methods used in classification rule training are illustrated in Fig. 2, using classification of tumor-infiltrating lymphocytes as an example. When making a prediction, many classification rules also produce a confidence measure that represents the expected accuracy of this prediction. In active learning, low confidence objects are labeled to fill gaps in the training set to improve accuracy. Given a classification rule, the set of unlabeled objects are first classified to generate prediction confidences. Labels are then solicited for low confidence objects, and these objects are added to the training set to re-train the classification rule. Figure 2A illustrates a classification rule as a partition of the histomic feature space into region corresponding to distinct cytologic classes. Feature values determine the positions of objects in this space, with classifications being less certain approaching the partition boundary. By iterating between labeling low confidence objects and re-training the classification rule, a feedback loop is established with the user to build a comprehensive training set that increases expected prediction accuracy. This label-update-predict cycle is repeated until the desired performance is achieved. Our software currently uses random forests as the classification algorithm, however other algorithms that provide a measure of prediction confidence such as boosting, support vector machines, or neural networks could be utilized (see Figure S4 and Methods).

HistomicsML uses two active learning methods to solicit labels: 1. Instance-based and 2. Heatmap-based. Instance-based learning presents the user with 8 of the least confident objects with and array of thumbnail images that can be labeled. Clicking an instance/thumbnail will direct the whole-slide viewer to the location of this object so that the surrounding tissue context can also be visualized. In heatmap-based learning, color-coded heatmaps representing prediction confidence are generated for each whole-slide image, enabling users to zoom into “hotspot” regions that are enriched with low-confidence objects where they can quickly label many objects. Slides with hotspots can be identified using an image gallery where slides are sorted based on confidence statistics. Users can determine if more training is needed by browsing this image gallery to assess algorithm performance.

HistomicsML also provides interfaces for generating ground-truth datasets, for validating classifier accuracy, and for collaborative review of user annotations. The validation interface provides a whole-slide image viewer similar to the training interfaces that can be used to browse slides and to label objects to create independent validation datasets. These validation datasets are stored on the server and available by drop-down menu so that users can resume labeling or share these datasets with other users. A validation interface allows users to apply classifiers to a validation dataset to measure accuracy and AUC. A review interface was also developed that allows users to easily examine and revise label data (either training or validation sets). This interface organizes data by slide, presenting thumbnail images for each labeled object organized into columns by class label. Users can drag-and-drop these thumbnails from one class or another to change their label, or place them in an ignore category to remove them from the set. Since the thumbnail images are small, clicking on the thumbnail image will navigate the whole-slide viewer to the location of this object so that the surrounding tissue area can be examined. Interfaces are also provided for importing new datasets and exporting results for further analysis and integration with other tools. To simplify deployment, a pre-built Docker container is provided (https://hub.docker.com/r/histomicsml/active/). This container is platform-independent, and allows users to run HistomicsML on any system without building the project from source, avoiding the need for installing library dependencies. Documentation on installation and use is also provided http://histomicsml.readthedocs.io/.

### Fast and accurate classification of vascular endothelial cells in gliomas

**Figure 3:**
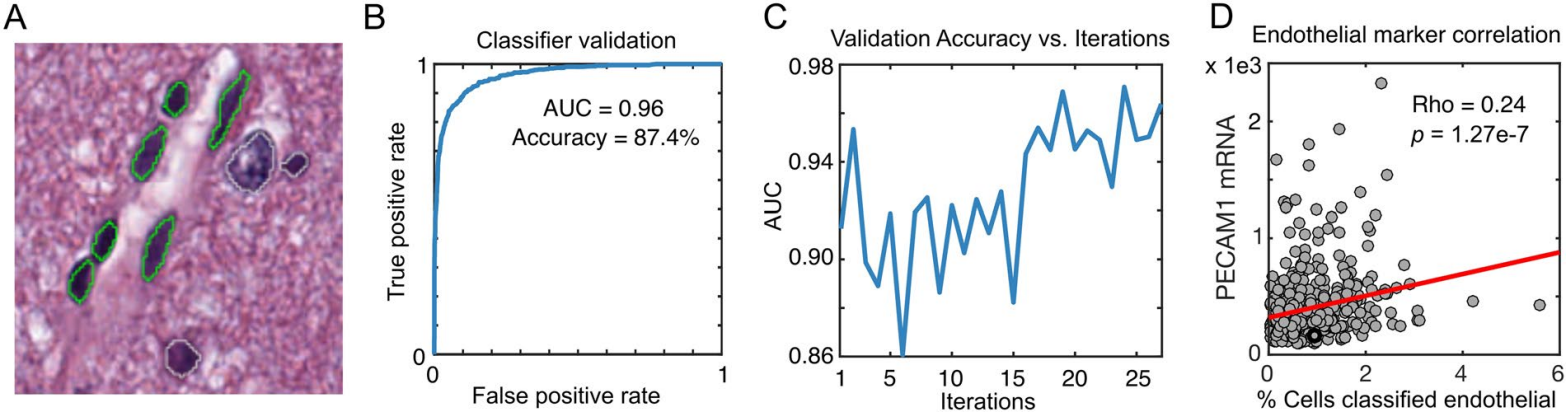
Classifying vascular endothelial cells in gliomas. (**A**) We used active learning to train a classification rule to identify vascular endothelial cell nuclei in lower-grade gliomas (highlighted in green). (**B**) Prediction rule accuracy was evaluated using area- under-curve (AUC) analysis. (**C**) AUC was evaluated at each training iteration to measure improvement in prediction accuracy. (**D**) For additional validation, we correlated the percentage of positively classified endothelial cells in each sample with mRNA expression levels of the endothelial marker *PECAM1* using measurements from TCGA frozen specimens (image analysis measurements were performed in images of formalin-fixed paraffin embedded sections from the same specimens).

We used the histomics toolkit (HistomicsTK, http://github.com/DigitalSlideArchive/HistomicsTK) to generate features for 360 million cell nuclei using 781 images (464 tumors) from The Cancer Genome Atlas Lower Grade Glioma (LGG) project. We trained a classification rule to identify vascular endothelial cell nuclei (VECN) and validated its performance using 67 slides not used in training (see Fig. 3, Figure S5). The VECN classifier was initialized by manually labeling 8 nuclei, and refined with both instance-based and heatmap-based learning to label 135 nuclei in 27 iterations. The VECN classifier is highly sensitive and specific, achieving an area-under-curve (AUC) of 0.964 and improving over the initial rule with AUC = 0.9234.

To further validate our VECN classifier, we correlated mRNA expression of the endothelial marker *PECAM1* with the fraction of cells classified as VECs in each specimen. *PECAM1* expression was significantly positively correlated with Percent-VECN (Spearman rho = 0.24, **p** = **1.27e-7**). We note that the mRNA measurements originate from frozen materials where image analysis was performed on fixed and paraffin embedded tissues that originate from same primary tumor but with unknown proximity to the mRNA sample.

To evaluate system responsiveness, we measured the time required for the update-predict cycles. We evaluated various sized datasets ranging from 10^6^–10^7^ objects (see Table S2). We observed a consistent linear increase of 1 second per 5.5 million objects on our 24-core server. This translates to a 10 second training cycle for a 50 million-object dataset.

### Phenotyping microvascular structures in gliomas

**Figure 4:**
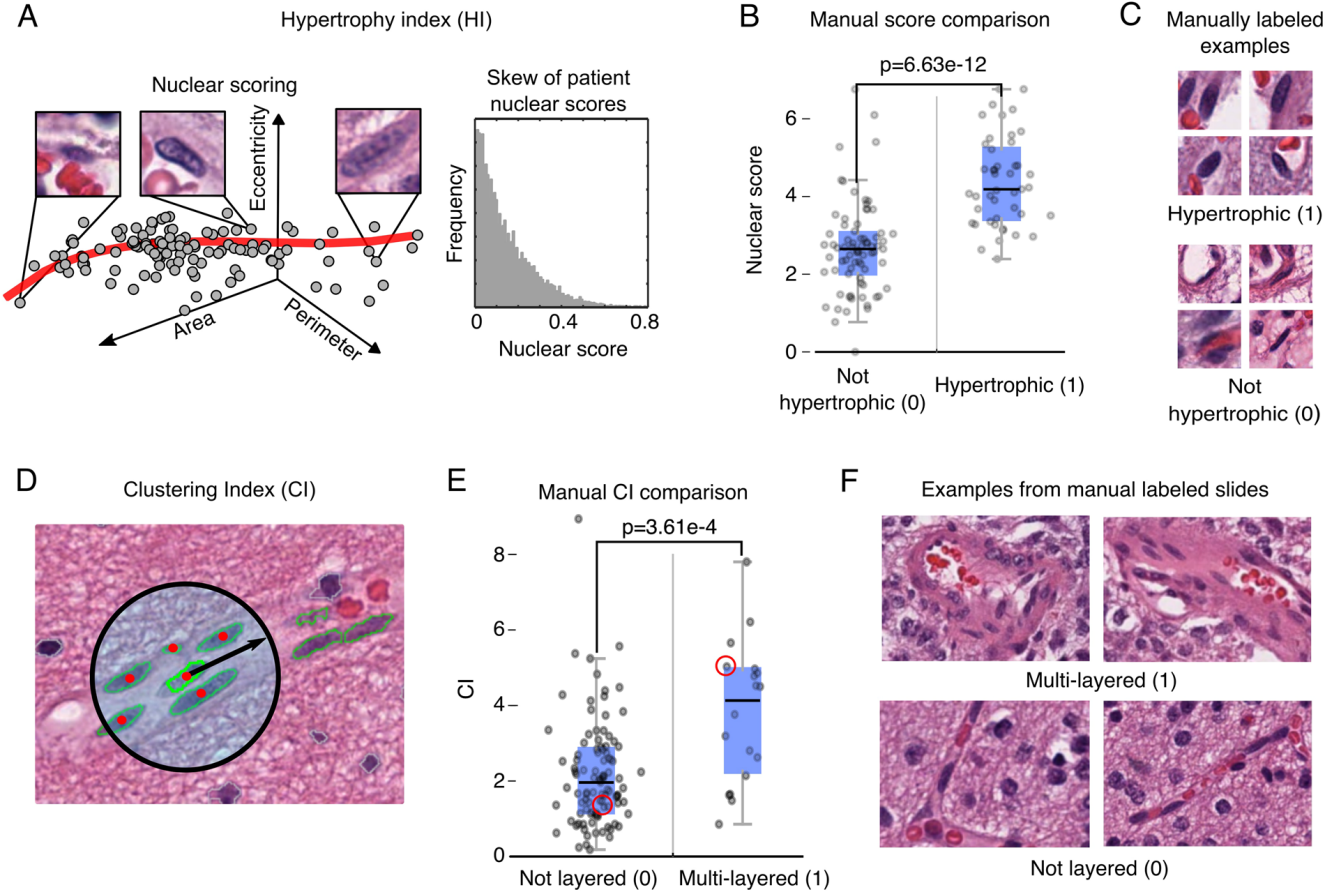
Quantitative phenotyping of microvasculature in gliomas. Microvascular structures undergo visually apparent changes in response to signaling within the tumor microenvironment. (**A**) We measured nuclear hypertrophy using a nonlinear curve to model the continuum of VECN morphologies. A hypertrophy index (HI) was calculated for each patient to measure the extremity of VECN nuclear hypertrophy score values. (**B**) We validated nuclear scores using nuclei that were manually labeled nuclei as hypertrophic/non- hypertrophic. (**C**) Examples of cell nuclei used in validation. (**D**) We implemented a clustering index (CI) to measure the spatial clustering of VECN as a readout of hyperplasia. CI measures the average number of VECN within a 50-micron radius of each VECN in a sample. (**E**) CI was compared to manual assessments of hyperplasia a multi-layered/not layered (red circles indicate the examples shown in F). (**F**) Example microvascular structures from two of the slides used in validating CI.

After demonstrating accurate VECN classification, we developed and validated quantitative metrics to describe the phenotypes of microvascular structures (see Fig. 4). Gliomas are among the most vascular solid tumors, with microvascular structures undergoing apparent transformations in response to signaling from neoplastic cells. *Microvascular hypertrophy*, or thickening of microvascular structures, represents an activated state where endothelial cells exhibit nuclear and cytoplasmic enlargement due to increased transcriptional activity. *Microvascular hyperplasia* represents the accumulation, clustering and layering of endothelial cells due to their local proliferation. While microvascular changes are understood to accompany disease progression, the prognostic value of quantitating their phenotypes in gliomas has not been established in the era of precision medicine, and may be beyond the capacity of human visual recognition.

Nuclear hypertrophy was scored using a nonlinear model to represent the continuum of VECN morphologies (see Methods). Nuclear scores were validated using 120 manually labeled VECN (45 hypertrophic, 75 non-hypertrophic) to show that nuclei labeled as hypertrophic score significantly higher (Wilcoxon **p** = **8.63e-12**). A hypertrophy index (HI) was then calculated to summarize hypertrophy at the patient level (see Methods). Hyperplasia was measured using a clustering index (CI) to capture the extent of proliferation and spatial clustering of VECN. CI was calculated at the patient level as the average number of VECN within a 50-micron radius centered at each VEC nucleus. CI was also compared to manual slide-level assessments of microvascular proliferation in 137 slides (18 presenting a multilayered phenotype) to show that images where multilayered structures are present associate with higher CI values (Wilcoxon **p** = **3.61e-4**).

### Microvascular phenotypes predict survival

Diffuse gliomas are the most common adult primary brain tumor and are uniformly fatal. Survival of patients diagnosed with infiltrating glioma depends on age, grade and molecular subtypes that are defined by *IDH* mutations and co-deletion of chromosomes 1p and 19q^26^. The lower grade gliomas (grades II, III) exhibit remarkably variable survival ranging from 6 months to 10+ years. Aggressive IDH wild-type (IDHwt-astrocytoma) gliomas having an expected survival of 18 months, where patients with gliomas having IDH mutations and 1p/19q co-deletions (oligodendroglioma) can survive 10+ years. Gliomas with IDH mutations but lacking co-deletions (IDHmut-astrocytoma) have intermediate outcomes with survival ranging from 3–8 years. The accuracy of grade in predicting outcomes varies depending on subtype^27^.

**Figure 5:**
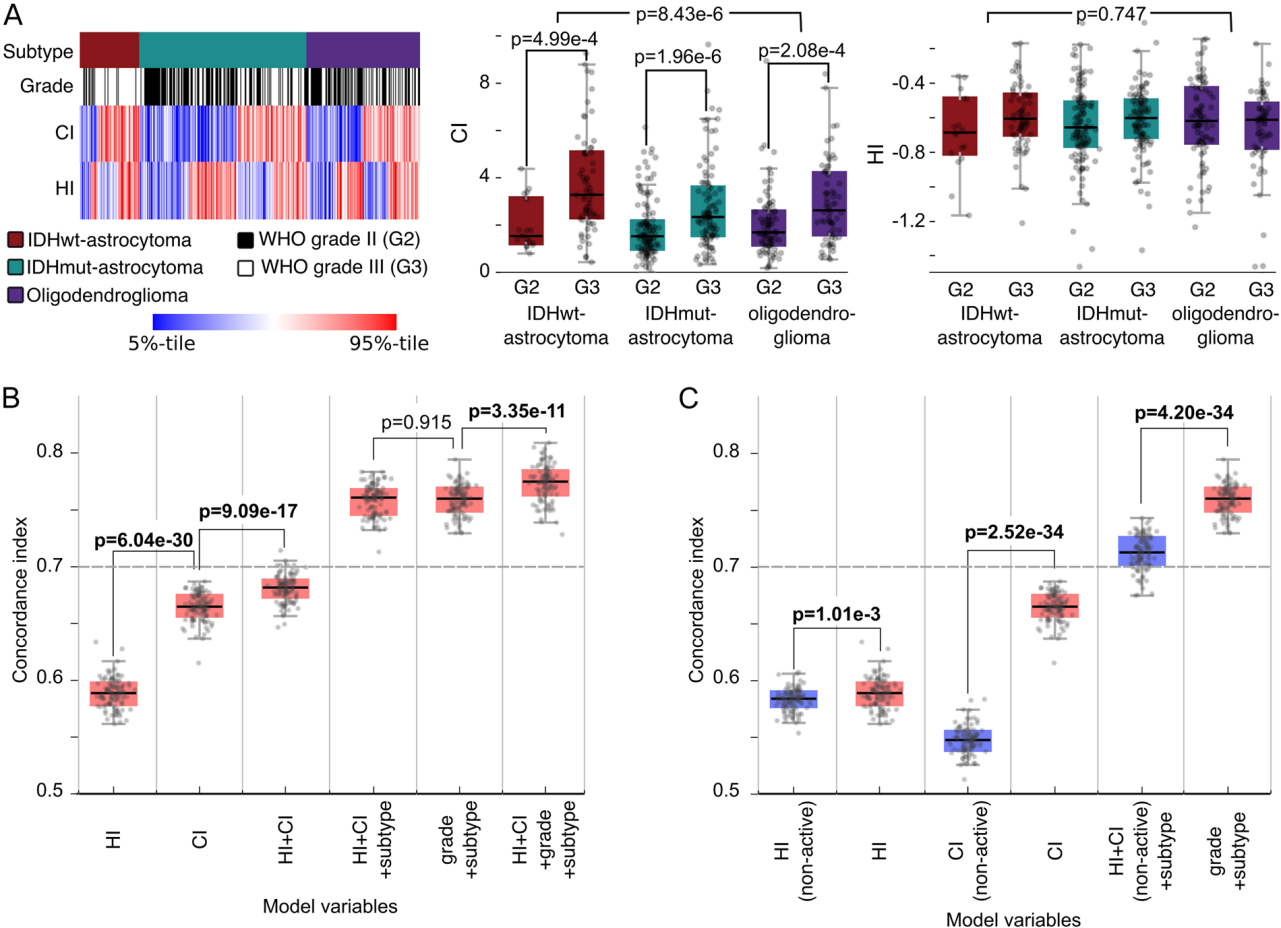
Predicting survival of glioma patients with microvasculature phenotypes. (**A**) HI and CI were compared with important clinical metrics including WHO Grade and molecular subtype. (**B**) We trained cox hazard models using combinations of phenotypic and clinical predictors to assess their prognostic relevance and independence. Models were trained and evaluated using 100 randomizations of samples to training/testing sets. The dashed line represents the c-index corresponding to molecular subtype in this cohort. (**C**) We compared the accuracy of models based on HI and CI generated using a classifier trained with active learning (red) with HI and CI generated using a standard classifier trained without active learning (purple).

We first investigated associations between hyperplasia and hypertrophy, grade and molecular subtype in the TCGA cohort using CI and HI (see Fig. 5A and Table S1). We found that IDHwt-astrocytomas exhibit a greater degree of microvascular hyperplasia than the less aggressive subtypes (Kruskal-Wallis **p** = **8.43e-6**), and that increased hyperplasia is also associated with higher grade within each molecular subtype (Wilcoxon IDHwt-astrocytoma **p** = **4.99e-4**, IDHmut-astrocytoma **p** = **1.96e-6**, oligodendroglioma **p** = **2.08e-4**). While differences in microvascular hypertrophy across subtypes and grades were not statistically significant (Wilcoxon **p** = **0.747**), the median HI for grade III disease was higher within each subtype. We also explored subtypes by using median CI or HI values to stratify patients into high/low risk groups (see Figure S6). Kaplan-Meier analysis found these “digital grades” were marginally prognostic in oligodendrogliomas (log-rank CI **p** = **6.87e-2**, HI **p** = **5.09e-2**) and IDHwt astrocytomas (CI **p** = **4.68e-2**), but remarkably neither CI nor HI could discriminate survival in the IDHmut-astrocytomas. Similar discrimination patterns were observed when stratifying by WHO grade.

After investigating associations with grade and subtype, we used a modeling approach to evaluate the prognostic value of microvascular phenotypes. Cox hazard models were created with various combinations of predictors including grade, subtype, CI and HI (see Fig. 5B). Patients were randomly assigned to 100 non-overlapping training/validation sets, and each was used to train and evaluate a model using Harrell’s concordance index (see Methods). Although HI-only models perform only slightly better than random (median c-index 0.58), HI+ CI models perform significantly better than CI-only models (**p** = **9.09e-17**). HI+ CI provide prognostic value independent of molecular subtype, improving the subtype c-index from 0.70 to 0.76. HI+ CI also performs as well as grade when combined with subtype (**p** = **0.915**), even though grade incorporates many more histologic criteria than microvascular appearance. Finally, HI+ CI also have prognostic value independent of grade+ subtype, increasing median c-index to 0.78 (Wilcoxon **p** = **3.35e-11**).

### Active learning training improves prognostication

To evaluate the benefit of active learning training, we repeated our experiments using a classification rule trained with a standard approach where the expert constructs a training set without the aid of active learning feedback. Using the same image collections described above, 135 cell nuclei were labeled in the training images (roughly evenly split between VECN and non-VECN). A classification rule was trained using these labels and applied to the dataset to compare classification and prognostic modeling accuracies with the active learning classifier.

The validation AUC of the standard classifier was 0.984 (AUC = 0.964 for active learning classifier). While the AUC measured on the validation set was higher, the standard learning classifier is much less specific on the entire dataset, producing very high estimates of percent-VECN in the TCGA cohort ranging from 7.1–57.2% (compared to 0.02–5.6% percent-VECN for active learning). Agreement between *PECAM1* expression and percent-VECN was much lower for the standard classifier percent-VECN (Spearman rho = 0.16 versus 0.24). We calculated updated HI and CI metrics using the standard classifier results and found that prognostic models based on these metrics were no longer predictive of survival (see Fig. 5C). The median c-index of models based on CI alone fell to <0.55 (Wilcoxon **p** = **2.52e-34**). Models incorporating HI+ CI+ subtype were also no longer equivalent to subtype+ grade models (**p** = **4.20e-34**), and only slightly better than subtype.

### Integrating phenotypic measures with genomic information

The molecular mechanisms of angiogenesis in gliomas have been studied extensively, and are targeted through anti-VEGF therapies like Bevacizumab^28^. To investigate the molecular pathways associated with CI/HI, we performed gene-set enrichment analyses^29^ to correlate CI and HI with mRNA expression. We analyzed IDHwt-astrocytomas and oligodendrogliomas separately since mechanisms may vary across subtype (IDHmut-astrocytomas were not analyzed). A partial list of pathways enriched at FDR q < 0.25 significance is summarized in Table 1 (extended results in Table S2).

**Table 1:** Molecular pathways enriched with phenotype-correlated transcripts.

Pathway Group	Pathway name	Leading-edge genes	Subtype/metric (directionality)	Nominal p-value (FDR q-value)
Classiscal angiogenesis pathways	*HIF1-alpha transcription factor	*PFKL, PFKFB3, ALDOA, PGK1, HK1*	Oligodendroglioma/HI (+) Oligodendroglioma/CI (−)	0.033 (0.179) <0.001 (0.116)
	HIF2-alpha transcription factor	*VEGFA, VHL, ARNT*	IDHwt-astrocytoma/CI (+) Oligodendroglioma/CI (+)	0.004 (0.017) 0.024 (0.116)
	VEGFR1/2 mediated signaling	*BRAF, MAPK1/14*	IDHwt-astrocytoma/HI (+) Oligodendroglioma/CI (+)	0.012 (0.144) 0.009 (0.12)
	*VEGFR1 specific signals	*MAPK1, NRP1/2*	IDHwt-astrocytoma/HI (+)	0.007 (0.19)
	Angiopoeitin receptor TIE-2 mediated signaling	*MAPK1/14, NFKB1, PIK3C*	IDHwt-astrocytoma/HI (+) IDHwt-astrocytoma/CI (+) Oligodendroglioma/CI (+)	0.014 (0.147) 0.015 (0.063) 0.009 (0.087)
	*PDGFRA signaling	*PIK3CA, FOS, PDGFRA*	Oligodendroglioma/HI (+)	0.014 (0.08)
Developmental signaling pathways	Notch signaling network	*NOTCH1, MAML1/2, MYC*	IDHwt-astrocytoma/CI (+) Oligodendroglioma/CI (+)	<0.001 (<0.001) 0.02 (0.146)
	*Notch mediated HES/HEY network	*HEY1, NOTCH1, MAML1/2, HIF1A*	IDHwt-astrocytoma/HI (+) IDHwt-astrocytoma/CI (+)	0.007 (0.143) <0.001 (<0.001)
	*WNT signaling	*WNT3A, GSK3B*	Oligodendroglioma/CI (+)	0.021 (0.085)
	*Regulation of nuclear beta catenin signaling		Oligodendroglioma/CI (+)	0.004 (0.054)
	*GLI-mediated Hedgehog signaling		IDHwt-astrocytoma/CI (+)	0.015 (0.063)
Other pathways	*Regulation of SMAD2/SMAD3 signaling	*SMAD3/4, MAPK1, MAP3K1*	IDHwt-astrocytoma/HI (+) IDHwt-astrocytoma/CI (+)	0.002 (0.008) 0.024 (0.139)
	*SMAD2/SMAD3 nuclear signaling	*SMAD3/4, CDK2/4, CDKN1A, AKT1, MYC*	IDHwt-astrocytoma/CI (+)	<0.001 (<0.001)
	*FOXM1 transcription factor network	*FOXM1, GSK3A, MYC, FOS*	Oligodendroglioma/CI (+)	<0.001 (<0.001)

Gene set enrichment analysis of the correlations between HI/CI and gene expression identified multiple pathways associated with gliomas and vascularization. Many of the significantly enriched pathways are specific to one molecular glioma subtype. Extended results are presented in Table S2.

Given the association between angiogenesis and hypoxia, we anticipated pathway analysis to identify strong relationships between microvascular phenotypes and classic hypoxia and metabolic glycolysis pathways. We found both HIF2A and VEGFR1/2 mediated signaling pathways were both upregulated with increasing CI and HI. Among the most strongly correlated genes were those involved in hypoxia and angiogenesis including *VEGFA*, *VHL*, *ARNT*, *PGK1* ^30^, *ADM* ^31^, and *EPO*, as well as glycolytic response mediators *HK1*, *PGK1*, *ALDOA*, *PFKFB3*, *PFKL* and *ENO1*. Angiopoietin receptor^32^ and Notch signaling^33^ pathways were also significantly enriched in both glioma subtypes.

Pathways with enrichment specific to IDHwt-astrocytomas included Notch mediated regulation of *HES*/*HEY* ^34^, GLI-mediated hedgehog signaling^35^, and SMAD signaling^36^, all of which have been linked to angiogenesis or regulation of structure and fate in vascular endothelial cells. Pathways with enrichment specific to oligodendrogliomas included WNT and beta-catenin signaling, and PDGFRA signaling (PDGFRA amplification is frequent in oligodendrogliomas).

We note that angiogenesis generally accompanies disease progression in gliomas, and that pathway enrichments may reflect molecular patterns associated more generally with disease progression in addition to angiogenesis-related microenvironmental signaling.

## Discussion

HistomicsML addresses the unique challenges presented by the scale and nature of whole-slide imaging datasets to enable investigators to extract phenotypic information. It is open-source and is available as a software container for easy deployment.

The endothelial cell classifier trained with active learning was highly accurate (AUC = 0.964), despite labeling only 135 cell nuclei. Although the amount of training data will vary depending on application, the web-based interface and active learning framework provided by HistomicsML significantly reduces the effort required to collect training data. The visualization and learning capabilities enable experts to rapidly label objects and to re-train and review classification rules in seconds. The web-based interface provides remote access to terabyte datasets, and enables fluid and seamless display of image analysis boundaries and class predictions associated with 10^8^+ histologic objects. Active learning directs labeling by guiding users to examples that provide the most benefit for classifier training, and improves efficiency by avoiding labeling of redundant examples.

Phenotypic metrics obtained using our endothelial classifier were validated using human annotations, and able to accurately predict survival of lower-grade glioma patients. We identified significant associations between microvascular phenotypes, grade, and recently defined molecular subtypes of gliomas. These investigations are timely in the current era of precision medicine, in which prognostic biomarkers have not been established within newly emergent genomic classifications of cancers. While it has long been established that angiogenesis is related to disease progression in gliomas, we showed that HistomicsML can be used to precisely measure subtle changes in microvasculature that perform as well as grade in predicting survival. Active learning was shown to both improve prognostication and agreement between histologic and molecular markers of VECNs in these experiments. The benefits of active learning have been shown to vary significantly depending on application, and in our experiments, the AUC of the active learning classifier was lower than the classifier produced by standard training (AUC 0.964 versus 0.984). Despite this, the prognostic measures derived from the active learning classifier had significantly better performance. One issue in evaluating active learning methods is the subjectivity involved with creating a ground-truth dataset. Free selection of ground-truth data mirrors the procedure for training a classifier without active learning, and so this ground-truth may not be a reliable measure of the benefits of active learning. This motivated us to look at more objective endpoints like patient survival or molecular information.

Integrating phenotypic metrics with genomic data identified recognized molecular pathways associated with angiogenesis and disease progression. These analyses are a template for how HistomicsML can link histology, clinical and genomic data to explore the prognostic and molecular associations of histologic phenotypes in other diseases. Histology contains important information that can be difficult or impossible to ascertain through genomic assays. Recent developments in the deconvolution of gene expression data can accurately estimate the proportions of cell types in a sample, but these approaches cannot provide spatial or morphologic information that often contains considerable prognostic or scientific value.

The software and experiments described in this paper have some important limitations. Our software currently does not generate the image segmentation or feature extraction data. The performance of segmentation algorithms is highly tissue-specific, and so segmentation algorithms should be tuned for each application. We provide links to tools for generating this data, but analysis of large histology datasets may require cloud or cluster computing resources. HistomicsML is interoperable with any image segmentation and feature extraction algorithm, and most classification algorithms, but our experiments only evaluated data from a single segmentation with a single feature set and classification method. Regarding scalability, the memory footprint of feature data is currently a limitation on the scale of datasets. In future versions, we plan to improve memory management, and to utilize commodity graphics processors to enable better scalability. Inter-reader variation is a significant issue in pathology, and although our software enables collaborative review of annotations, we do not yet have a systematic approach for integrating and filtering annotations from multiple users. Annotations were performed on hematoxylin and eosin stained sections, where cell type cannot be confirmed with absolute certainty. Future applications will explore the use of immunohistochemical staining for validation.

## Methods

### Software

Documentation for installing and using the software is available at http://histomicsml.readthedocs.io/en/latest/. Source code for the active learning system is published under the Apache 2.0 license at (https://github.com/CancerDataScience/HistomicsML). A Docker software container is also available for easy deployment (https://hub.docker.com/r/histomicsml/active/). This Docker container contains sample data for the whole-slide image depicted in Fig. 2.

### Data

Whole slide images, clinical and genomic data were obtained from The Cancer Genome Atlas via the Genomic Data Commons (https://gdc.cancer.gov/). Images of formalin-fixed paraffin-embedded “diagnostic” sections from the Brain Lower Grade Glioma (LGG) cohort were reviewed to remove images of sections with tissue processing artifacts including bubbles, section folds, pen markings and poor stain quality. For this paper a total of 781 whole-slide images were analyzed. Genomic data (described below) was derived from frozen materials from the same specimens. The relationship of diagnostic sections and frozen materials is unknown, other than that they originate from tissues produced during the same surgical resection.

Genomic and clinical data were acquired using the TCGAIntegrator Python interface (https://github.com/cooperlab/TCGAIntegrator) for assembling integrated genomic and clinical views of TCGA data from the Broad Institute Genomic Data Analysis Center Firehose (https://gdac.broadinstitute.org/). The same genomic platforms were used across all experiments. Gene expression values were taken as RSEM values from the Illumina HiSeq. 2000 RNA Sequencing V2 platform. Genomic classifications for IDH/1p19q status were obtained from the Supplementary Material of ^37^.

### Image analysis segmentation and feature extraction

The software pipeline used to segment cell nuclei and measure their histomic features is shown in Figure S1. This pipeline utilizes algorithms provided by the HistomicsTK Python library for histologic image analysis (http://github.com/DigitalSlideArchive/HistomicsTK) to perform color normalization, nuclear masking and splitting, feature extraction, and database ingestion. Images were normalized to an H&E color standard using Reinhard normalization. Tissue pixels were first masked from the background using linear discriminant analysis and then the mean and standard deviation of the tissue pixels in the L*A*B color space were calculated. These moments were mapped to match the moments of a color standard image prior to inversion back to RGB color space. This color normalization process considerably improves the quality of subsequent image analysis steps, improving the consistency of segmentation results and image features. Whole-slide images were tiled into 4096 × 4096 pixel tiles and processed separately. Cell nuclei were highlighted using color deconvolution algorithm to digitally separate the hematoxylin and eosin stains. Hematoxylin images were masked to identify nuclear pixels using a combination of adaptive thresholding and morphological reconstruction to remove background debris. Closely packed nuclei were then split using a watershed segmentation applied to the laplacian-of-gaussian response of the hematoxylin image. Nuclei were described using 48 histomic features describing shape, intensity and texture. These features include eccentricity, solidity and fourier shape descriptors (shape), statistics of hematoxylin signal including variance, median, mean, min/max, kurtosis, skew and entropy (intensity) and statistics of hematoxylin intensity gradients (texture). Computation was carried out in a cluster-computing environment using Torque to distribute slides to different computing nodes. Nuclear boundaries were stored in a text-delimited format and ingested into a SQL database to drive the web-based interface. Features are stored in HDF5 format on a RAID array.

### Validation and training

We selected 67 slides from the LGG cohort to validate the performance of a vascular endothelial classifier. A field containing a mixture of nuclei from vascular endothelial cells and other cell types (tumor nuclei and inflammatory cells, for example) was selected in each slide. Each correctly segmented nucleus in the field was labeled as either vascular endothelial or “other”. Incorrectly segmented nuclei and nuclei that were too ambiguous to classify with a high degree of certainty were ignored. In total 2479 cell nuclei were labeled. Labels were reviewed by a board-certified neuropathologist. Classifiers were trained using a mixture of instance-based and heatmap-based feedback.

Annotations to validate hypertrophy index and clustering index were acquired by manual inspection of digital slide images by a board-certified pathologist who was blinded to the computer-generated HI and CI scores. A selection of 120 cell nuclei classified as VECN were labeled as either hypertrophic (45 nuclei) or non-hypertrophic (75 nuclei) using the HistomicsML Review tool. Nuclear hypertrophy scores were compared for these manually labeled nuclei using a non-parametric Wilcoxon sign rank test. For clustering index, 137 slides were manually reviewed to determine if they present microvascular hyperplasia and proliferation (multi-layered vessels). CI scores were compared for slides containing multi-layered vessels and slides not containing multilayered vessels using the Wilcoxon test.

### Machine learning

Random forest classifiers (OpenCV (v2.4.10)) were used due to their efficiency and resistance to overfitting. The random forest parameters are the number of trees (fixed at 100), maximum tree depth 10, and 7 features selected for node splits (selected as the square root of the number of features). Confidence for object *i* is calculated by tree votes

1 $${c}_{i}=| \sum _{j=1}^{N}{t}_{j}| ,{t}_{j}\in \{-1,1\},$$

where *t*_*j*_ is the prediction from tree *j* of *N* total trees. Minimum confidence is achieved with a 50/50 split. Calculations were threaded to maintain the responsiveness of the system when predicting datasets containing 10^7^+ objects.

### Clustering index

CI was calculated using a modified version of the Ripley’s K-function spatial statistic to capture the degree of “spread” of events in spatial domains^38^. Since microvascular hyperplasia increases in VECN density, we excluded Ripley’s density normalization terms. Edge-effect corrections were also ignored due to the extremely large number of objects scarcity of objects located at the tissue edges. CI was calculated as:

2 $$CI(\tau )=\frac{1}{K}\sum _{i=1}^{K}| {{\rm{\Omega }}}_{i}| ,\,{{\rm{\Omega }}}_{i}=\{{d}_{i,j}\le \tau \},\,{d}_{i,j}={\parallel {x}_{i}-{x}_{j}\parallel }_{2},$$

where *K* is the number of nuclei, *d*_*i,j*_ is the Euclidean distance between objects *i*, *j, τ* is the search radius. This effectively calculates the average number of objects within distance *τ* = 50-microns of each VECN in the slide.

### Hypertrophy index

A principal curve was trained to model the morphological continuum of VECN and then used to score the hypertrophy for each nucleus classified as VECN^39^. The principal curve models the feature vector values *f*_*i*_ of nucleus *i* as

3 $${f}_{i}=g({\lambda }_{i})+{e}_{i},$$

where *g* is a 1D nonlinear curve parameterized by *λ*, and *e*_*i*_ is the model error. The fitted principal curve is used to score each nucleus by projecting *f*_*i*_ onto the curve and calculating the path length *s*_*i*_ from the curve origin

4 $${s}_{i}={\int }_{{\lambda }_{0}}^{{\lambda }_{i}}\parallel g^{\prime} (z)\parallel dz$$

where *λ*_0_ is the origin, *λ*_*i*_ is the location of the least-squares projection, and *g’* is the curve tangent function. Hypertrophic VECN will have longer path length values and thus higher *s*_*i*_. The principal curve was constructed using histomic shape features for nucleus area, eccentricity and perimeter. The directionality for the beginning/end of the curve was established by initializing the curve fitting with a single normal appearing VECN and a single hypertrophic VECN.

A patient-level HI was calculated to represent the population-level skew of *s*_*i*_ towards hypertrophic morphologies in a slide. HI was measured as the negative skew of nuclear scores

5 $$SI=-\frac{1}{K}\sum _{i=1}^{K}{({s}_{i}-\bar{s})}^{3}{(\frac{1}{K-1}\sum _{i=1}^{K}{({s}_{i}-\bar{s})}^{2})}^{-3}$$

where *K* is the number of objects/nuclei in the image, *s*_*i*_ is the hypertrophy score of object *i*, and $\bar{s}$ is the mean of the hypertrophy score*s s*_*i*_.

### Pathway analysis

Spearman rank correlation was used to compare RNAseq and CI/HI values. We performed gene set enrichment analyses for CI/HI and subtype combinations. Gene symbols were harmonized to the HUGO Database (http://www.genenames.org/)^40^. Enrichment analysis of Spearman.rnk files was performed with the GSEAPreranked (v4.2) module in GenePattern with 1000 permutations. We tested enrichment for pathways described in the NCI/Nature Pathway Interaction Database (PID) using a version of the MSigDB (http://software.broadinstitute.org/gsea/msigdb) C2 Curated Gene Sets that was filtered to remove non-PID pathways. We reported both the nominal p-values as well as FDR-corrected q-values produced by GSEA in Table 1 and Table S2.

### Statistical analysis

HI/CI values were compared across subtype and grade using the Wilcoxon rank sum test for grade or the Kruskal-Wallis test for subtype. Survival differences were evaluated using the log-rank test. Classifier performance was reported as area under receiver operating characteristic curve with *p*_*i*_ values. Prognostic model performance was measured using Harrell’s concordance index^41^.

### Hardware

All studies were performed using a multi-socket multicore server equipped with two Intel Xeon e5–2680 v3 2.5 GHz processors, 128 GB memory, and 14 TB main disk storage in a RAID10 array.

### Data availability

This paper was produced using large publicly available image datasets. The authors have made every effort to make available links to these resources as well as making publicly available the software methods used to produce these analyses and summary information. All data not published in the tables and supplements of this article are available from the corresponding author on request.

## Electronic supplementary material

Supplementary Information

Supplementary Dataset 1

Supplementary Dataset 2
