# Supplementary material for: Interactive phenotyping of large-scale histology imaging data with HistomicsML
Source: Sci Rep. 2017 Nov 6;7:14588. doi: 10.1038/s41598-017-15092-3 (PMC5674015; doi:10.1038/s41598-017-15092-3)
Supplement: Supplementary file 1 — Supplementary Information [file 41598_2017_15092_MOESM1_ESM.pdf]

SUPPLEMENTARY INFORMATION

Interactive phenotyping of large-scale histology imaging data with HistomicsML

Michael Nalisnik[1], Mohamed Amgad[1], Sanghoon Lee[2], Sameer H. Halani[3], Jose Velazquez Vega[4], Daniel J Brat[4,5], David A Gutman[2], Lee AD Cooper[1,5,6]

[1]Department of Biomedical Informatics, Emory University School of Medicine
[2]Department of Neurology, Emory University School of Medicine
[3]Emory University School of Medicine
[4]Department of Pathology & Laboratory Medicine, Emory University School of Medicine
[5]Winship Cancer Institute, Emory University
[6]Department of Biomedical Engineering, Georgia Institute of Technology / Emory University School of Medicine
Atlanta GA, USA

* Correspondence: lee.cooper@emory.edu

SUPPLEMENTARY METHODS

User interface. HistomicsML is implemented as a web-based application using the Boostrap (v3.2.0) and Knockout (v3.1.0) libraries for dynamic UI updating. A viewer capable of panning and multi-resolution zooming of pyramidal image formats is implemented using IIPImage (v1.0, http://iipimage.sourceforge.net/) on the server side and OpenSeaDragon (v1.0.0, https://openseadragon.github.io/) on the client side. Whole-slide images, typically available in svs or ndpi formats, must first be converted to a non-proprietary pyramidal TIFF format using VIPS (v4.42.3, http://www.vips.ecs.soton.ac.uk/) and OpenSlide (v3.4.0, http://openslide.org/).

Image analysis can generate tens of millions of polyline annotations that delineate the boundaries of objects. The viewer can display these annotations in real-time by generating a scalable vector graphics (SVG) overlay in real-time. Boundary polyline annotations are stored in a MySQL database indexed by slide, x-centroid and y-centroid. As the user pans/zooms the viewer, the OpenSeadragon API generates magnification and position information for the current field of view. Annotations contained in the current field are queried from the database and an SVG document containing the polyline coordinates is dynamically generated. Panning and zooming events are used to scale/translate the SVG objects as the user changes the field.

A spatial caching scheme was implemented to ensure seamless display of annotations during panning. During the database query, the annotations located in the surrounding fields are also retrieved and generated in the SVG document. Even though these objects are not visible in the current field, they will be instantly visible upon panning without requiring an additional database query. Following a pan/zoom event a new SVG document is generated in the background without interrupting the display. Efficient database indexing is used to ensure rapid generation of the SVG in the background. The annotations are indexed first by image/slide, then by x-centroid location and y-centroid location. This ordering significantly accelerates the query since the annotations from all other slides are filtered first.

The viewer can display transparent heatmap overlays to illustrate the spatial patterns in classifier confidence or the density of positively classified objects. At low resolutions the visibility of individual objects is lost due to their small size, and so a visualization mechanism is needed to guide users to locations where cells of interest are located or where active learning feedback is desired. Given the current state of the classifier and the predicted class of all objects a JPEG heatmap is generated. Each whole-slide image is divided into a grid of 40 x 40 pixel cells at full magnification. For each grid cell, we identify the objects located in that cell, and calculate both the percentage of “positive” class objects and the maximum object classification uncertainty. Each of these images is smoothed with an 11 x 11 pixel gaussian filter with standard deviation 3.5, and then standardized to the range [0, 255] to generate an intensity image. The OpenCV library (v2.4.10) is used to perform operations necessary to generate the heatmaps.

Learning session database. Along with storing the object annotations the MySql database also organizes whole slide image files into datasets (e.g. by tissue type), links image files to their annotation metadata, and keeps track of existing learning sessions. For datasets the database has fields for image names, image dimensions and magnification, and the feature file associated with each images. For learning sessions the database stores the session name, class names, the dataset associated with the learning session, the selected training objects and their assigned labels, the active learning iteration where each object was labeled, and the filename of the HDF5 file containing the learning session.

Interface design. The entry page enables users to start a new learning session or to resume a previous learning session (see Figure S3A). To start a new session users can select from the available datasets from a drop-down menu, enter a name for the session, and enter the names of the positive and negative classes in the provided text fields. To initialize the classifier, the user is then directed to a “priming” screen to select 4 examples from each class. The priming screen contains a whole slide image viewer that displays the selected slide and boundary annotations. Users can select examples by double-click, which highlights their boundary in yellow, and adds a thumbnail image of the selected examples to an array above the viewer. Following this

labeling the initial classifier is trained and applied to the entire dataset to generate initial class predictions and confidence values. The user then enters the main active learning interface where they will provide additional labels through active learning feedback. For resuming a session users first select a dataset from a drop-down menu, and then a second drop-down is populated from the database with all existing sessions that are associated with that dataset. Selecting a session then launches the user directly into the main learning interface.

In the active learning session users can alternate between instance-based feedback and heatmap-based feedback screens. In the instance-based feedback page, 8 samples selected as “ambiguous” based on prediction confidence are displayed as an array of thumbnails above a viewer, each labeled with its predicted class (see Figure S3B). Clicking an example thumbnail will direct the slide viewer to focus on the slide/region surrounding this object (the object is highlighted in the center of the screen). Double clicking the thumbnail will toggle the label among the possible classifications (positive/negative/ignore). The ignore option is provided to remove examples that are improperly delineated or where the user is not able to label the object with certainty. Labeling an object with ignore removes it from the training set and from the pool of unlabeled data.

In the heatmap thumbnail gallery page, slides are displayed in a scrollable list overlaid with their heatmap and sorted by minimum average prediction confidence (to put slides enriched with informative examples near the top) (see Figure S3C). A user can click on a slide thumbnail in this gallery to navigate to the slide viewer where labeling feedback can be provided (see Figures S3D, S3E). This displays the slide in the whole-slide image viewer with the heatmap overlay which allows users to zoom into feedback areas at high magnification. Zooming to 10X magnification and beyond, the heatmap is replaced by the object annotations that are color coded by predicted class. To correct a misclassification, the user can double click within the object’s boundary to toggle the object class and to add this object to the training set. When done correcting errors a submit button will re-train the classifier.

In addition to the active learning interfaces, we provide a review page where the samples of the training set are displayed, organized by class and slide (see Figure S3E). This interface permits additional review of the labeled examples and enables the users to change labels using drag-and-drop. This features facilitates multiple reviewers for collaboration among less and more experienced reviewers.

Input / output data formats. Our system utilizes three input data formats: 1. Whole slide pyramidal TIFF images generated by VIPS 2. Object boundaries in a text-delimited format 3. Object features in HDF5 binary format. Images are converted from proprietary microscope vendor formats to a pyramidal TIFF format using VIPS and OpenSlide. Object boundaries are consumed as comma separated values into the MySQL database using the INFILE command. Histomic features are stored in the HDF5 facilitate efficient loading and to maintain internal organization of objects by patient and slide. Correspondence between object annotations and histomic features is maintained using database object IDs in the HDF5 files. In addition to the features and database IDs, the HDF5 files contains the object centroids, slide names, and normalization data used in z-scoring the feature values. For output formats, users can store trained classifiers in HDF5 format, capturing the name of the training set, the dataset from which it was created, the object database IDs, class labels of objects labeled during training, histomic features of training objects, and the iteration in which each object was added.

Command line tools. A command line tool for applying trained classifiers outside of the user interface is also provided. This tool provides enables users to perform prediction and quantification of large datasets offline after training a classifier. The command line tool takes as input a classifier HDF5 file and an HDF5 file of histomic features for objects to be classified (in the input format described above). The prediction function will generate a new HDF5 file that supplements the input file with predicted class labels and prediction confidence scores. The quantification tool provides basic quantification (counting) of objects in each slide, and generates a CSV file with the slide name, positive class count and negative class count for each slide present in the input HDF5 file.

SUPPLEMENTARY FIGURES AND TABLES

Table S1. Dataset summary (see Excel file).

Table S2. Gene set enrichment analyses (see Excel file).

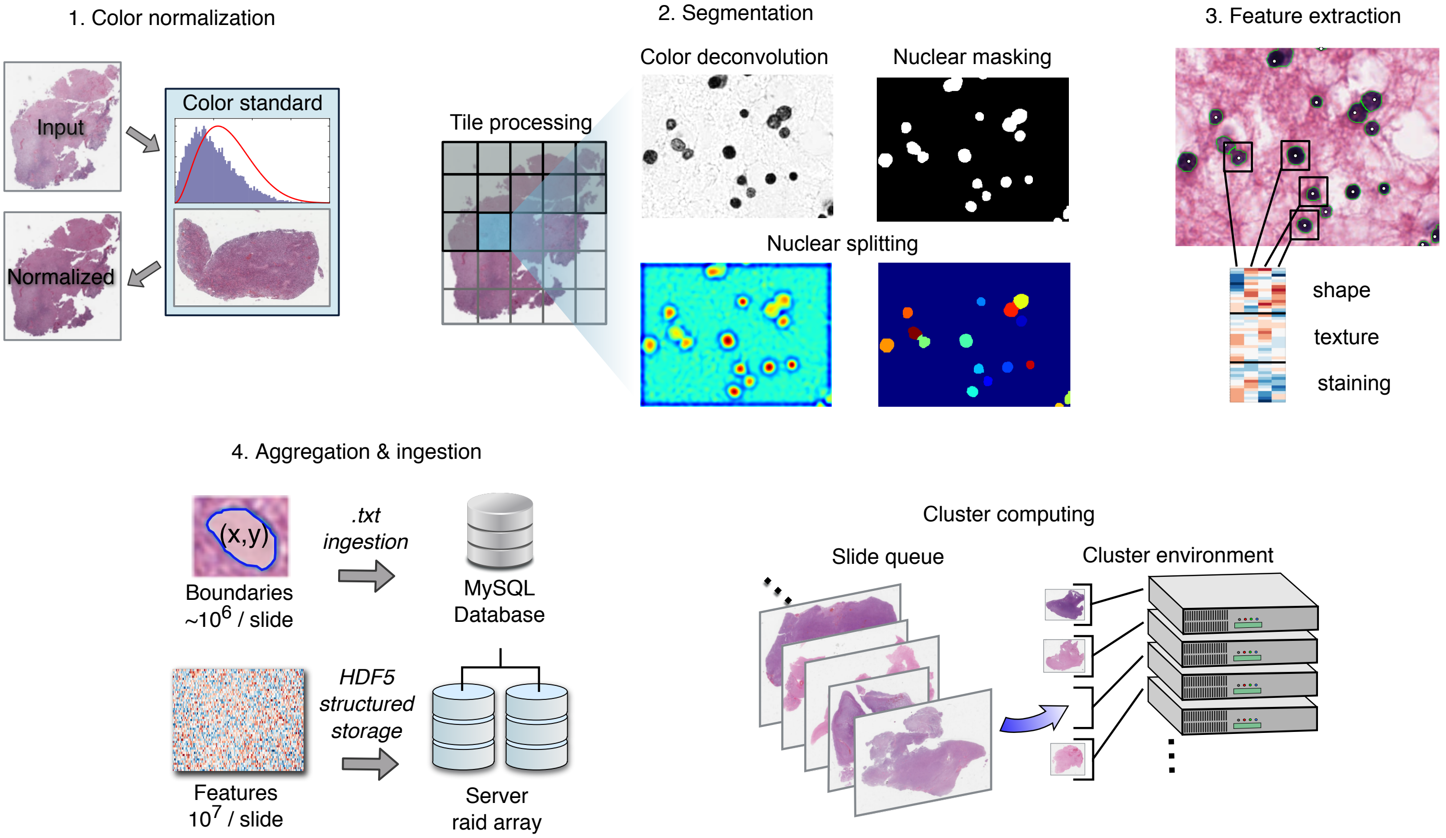

Figure S1. Image analysis pipeline. The studies presented in this paper used an image analysis pipeline for analyzing cell nuclei in whole-slide images based on HistomicsTK (https://github.com/DigitalSlideArchive/HistomicsTK), a software library for digital pathology image analysis. Step 1 in this pipeline normalizes the color characteristics of each slide to a gold standard H&E to improve color-deconvolution and downstream filtering operations. Step 2 processes the slide tile-wise, first digitally unmixing color images into eosin and hematoxylin stain images, then analyzing the hematoxylin image to mask nuclear pixels using a Poisson-Gaussian mixture model and smoothing this binary mask with a graph-cutting procedure. We then apply a constrained Laplacian of Gaussian filter to split closely packed cell nuclei. In step 3, a set of 48 features describing shape, texture and staining is calculated for each segmented cell nucleus. Finally, in step 4 all segmentation boundaries and features from each slide are aggregated into a single file. A delimited-text format is used for object boundaries, which are ingested into a SQL database to drive visualization in the user interface. Features are stored in a HDF5 structured format on a RAID array for fast and convenient access in training and evaluating classification rules.

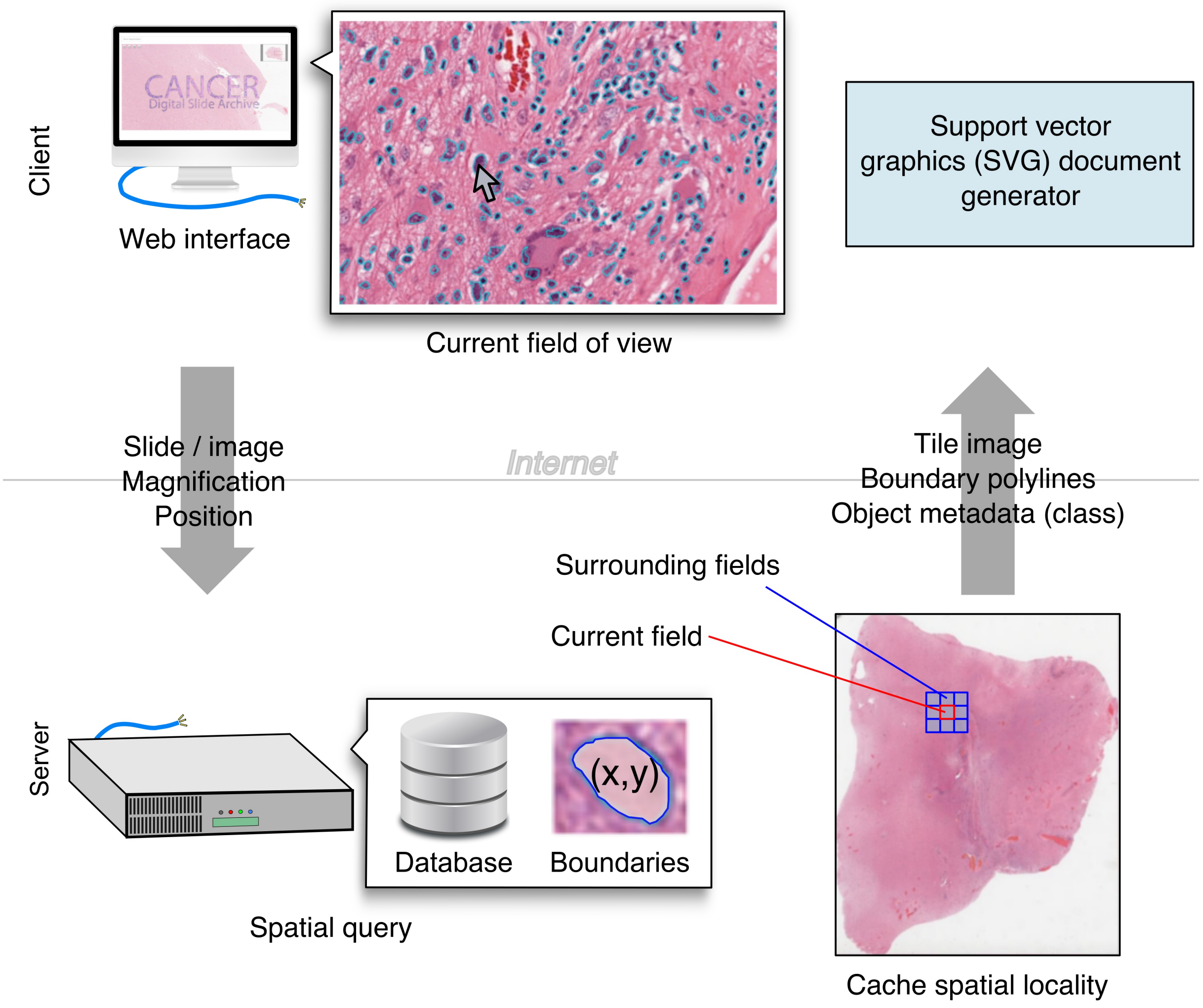

Figure S2. Scalable display of boundaries. Each whole-slide image can contain a million or more histologic entities, each with polygonal boundaries that consist of multiple (x,y) vertices. Rendering these boundaries fluidly requires effective database query, client-server communication and spatial caching. Our software framework renders boundaries in the web interface using a dynamic strategy outlined here. Following a mouse event, the current field of view (position/magnification) is communicated to the server. If the magnification is at or above 10X, the database is queried to identify objects in the current and adjacent fields. The image data, object boundaries and object metadata (including class) are communicated back to the web client. The web-client then constructs a Scalable Vector Graphics (SVG) document that contains the boundary polylines and that encodes any classification information using color tags. This strategy provides fluid visualization and does not incur any delay on a panning event in the client viewer, since the adjacent regions are already encoded in the SVG document.

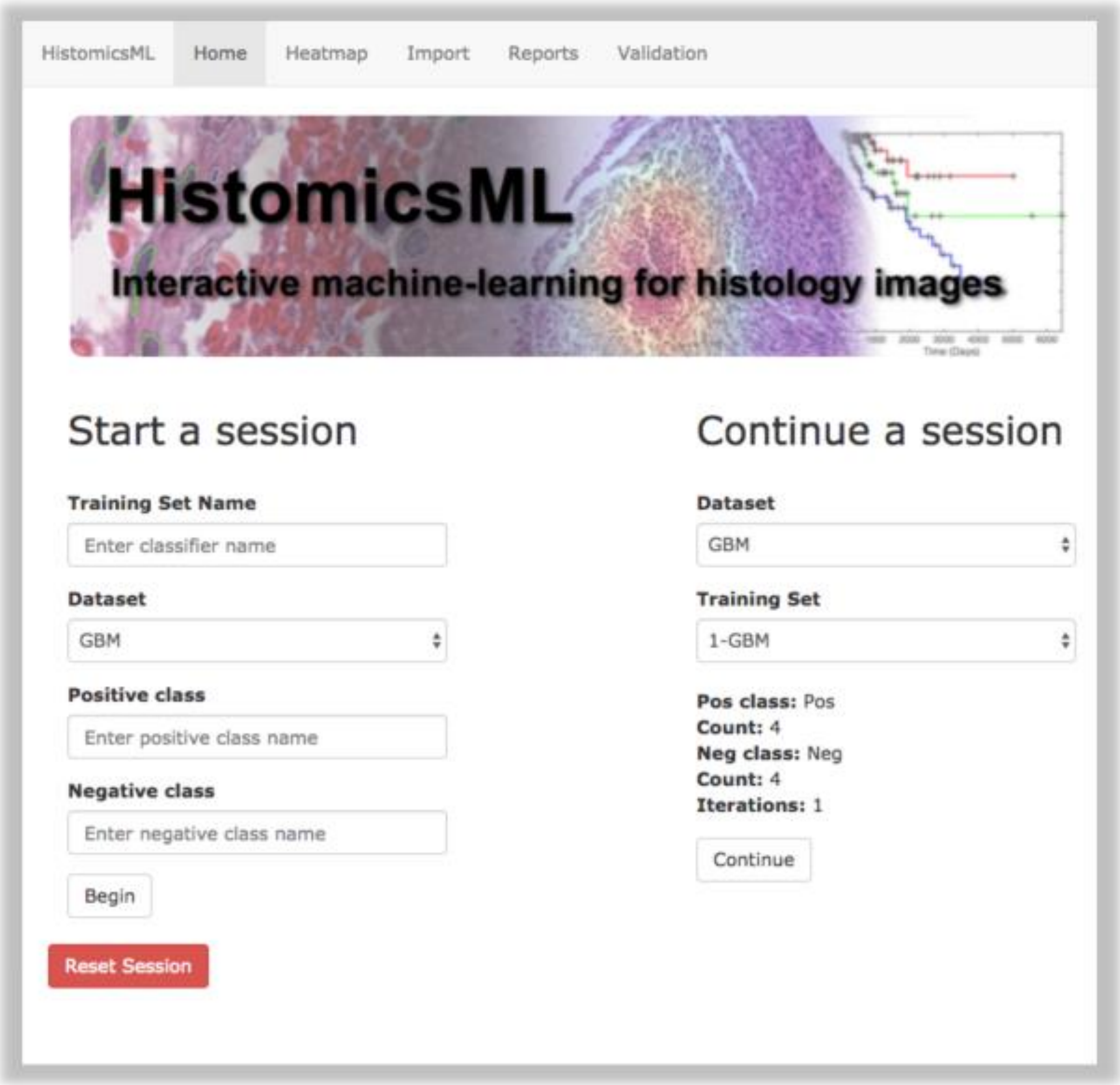

Figure S3A. Home interface. The landing page enables users to initiate a new learning session or to continue an existing session. For starting a new session users select a dataset, provide a session name and assign class names for training. Selecting a dataset from the continue session option populates a drop-down list displaying the session names, class names and labeled example statistics for sessions associated with that dataset.

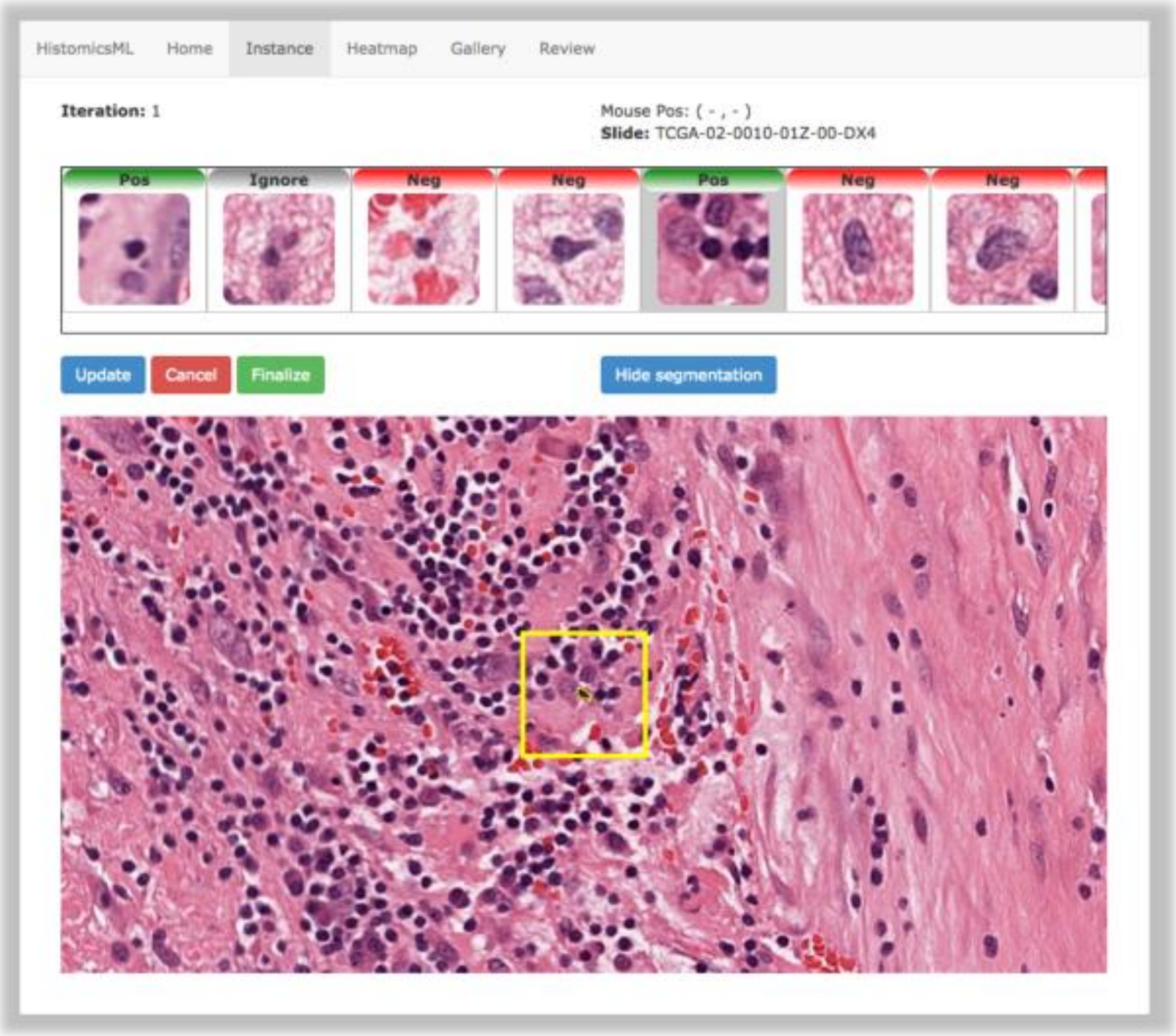

Figure S3B. Instance-based learning interface. This view facilitates the labeling of samples selected by active learning to refine the classification rule. Thumbnail images of 8 instances selected as valuable by active learning are displayed in an array along with their predicted class. Clicking a thumbnail directs the whole-slide image viewport to the slide/region surrounding this sample. Double-clicking the thumbnail image cycles the assigned class labels. After correcting errors the user can commit these samples to the training set and update the classifier. They can then resume with additional feedback or finalize the classification rule.

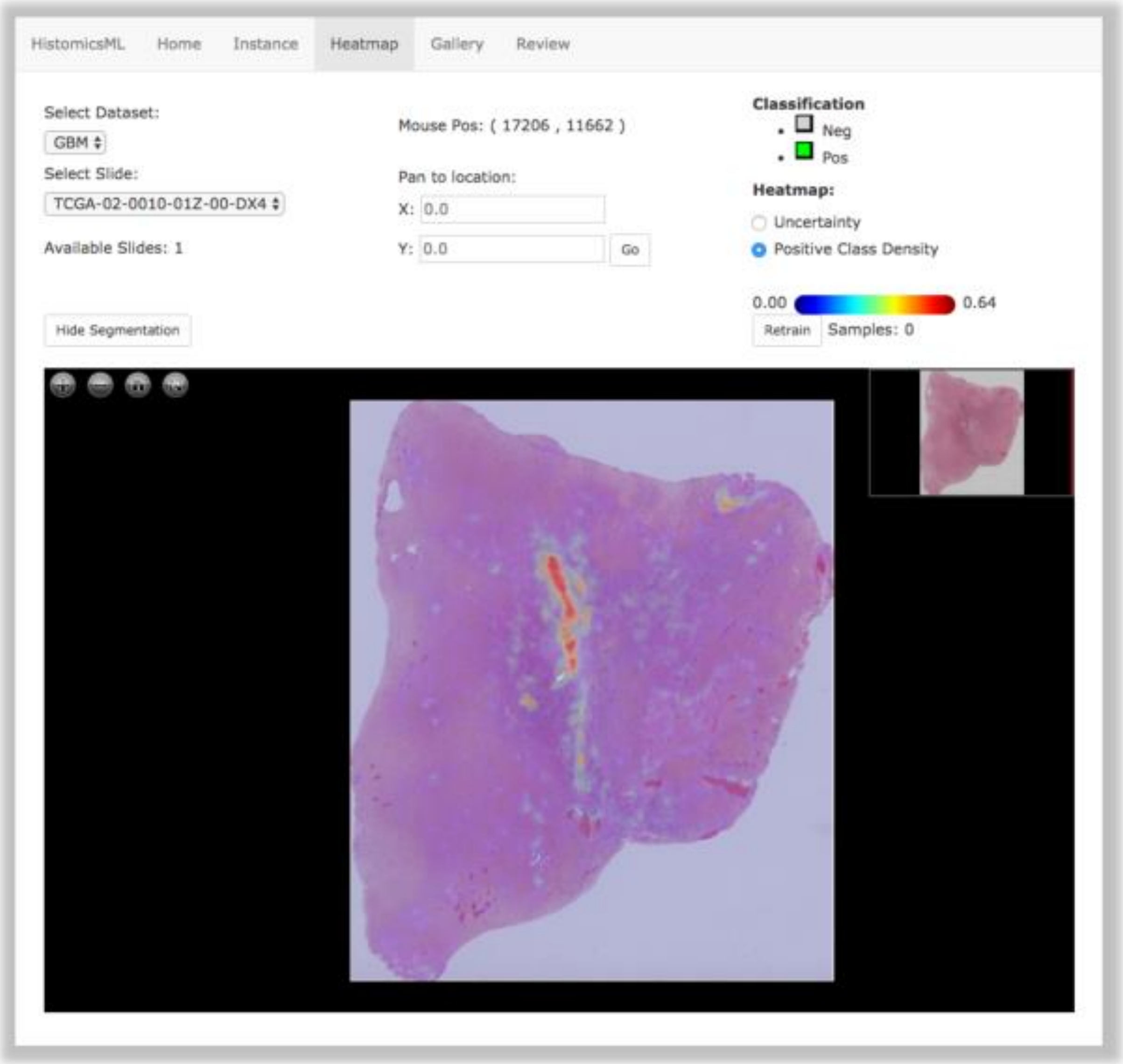

Figure S3C. Viewer-based learning interface. This view enables the overlay of heatmaps of classification confidence or positive class density in a whole-slide imaging viewport. Users can zoom into hotspots to review the classification rule predictions and to provide additional feedback in key regions that are likely to contain false positive or false negative predictions (see next panel).

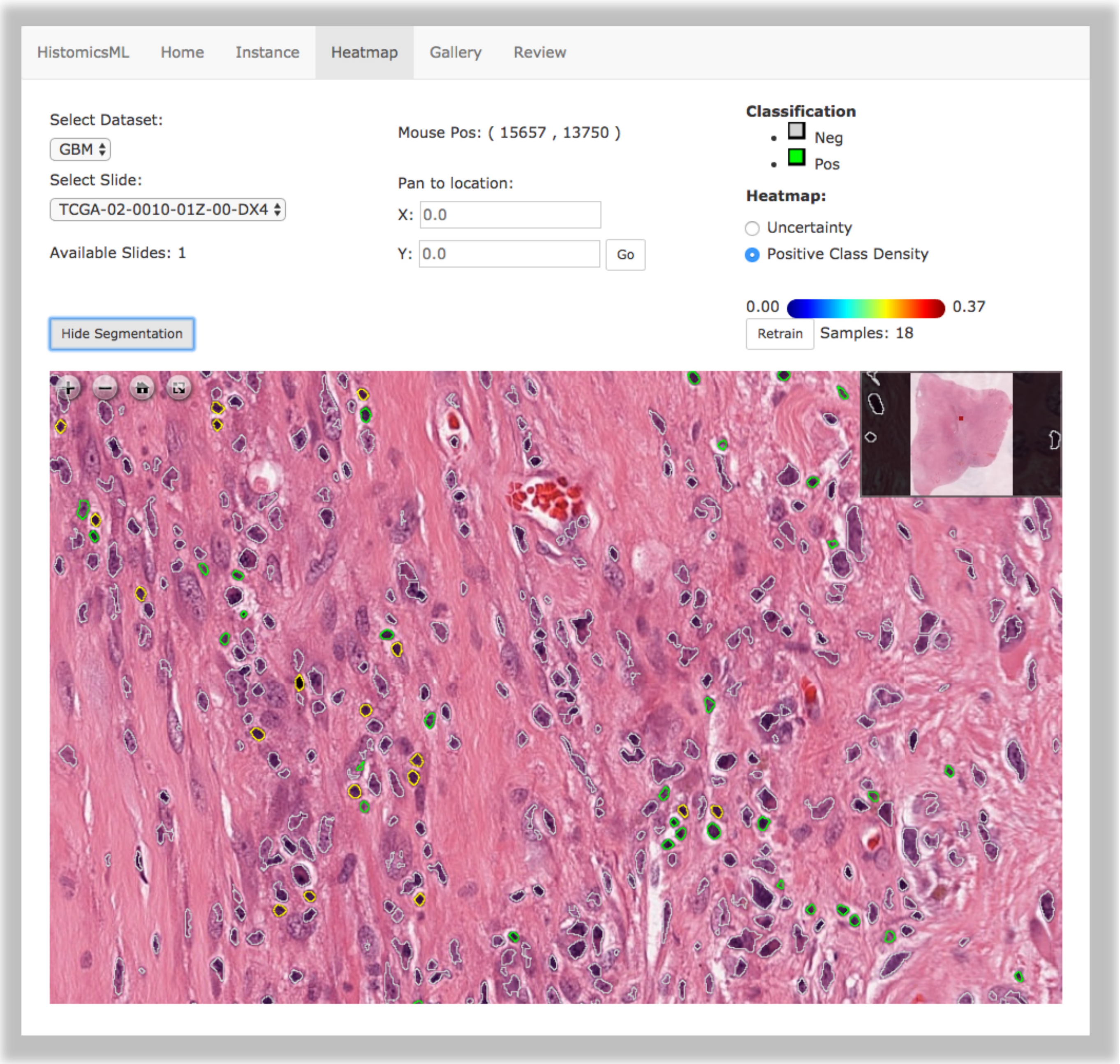

Figure S3D. Viewer-based learning interface (zoomed). Zooming into a hotspot region users can review and correct predictions for individual objects. Here cell nuclei that are positively classified are indicated with green boundaries and others indicated with white. Users can single-click objects in this view to correct prediction errors (yellow) - cycling their class label and committing them to the training set. The classifier can also be updated from within in this view to visualize the results of feedback.

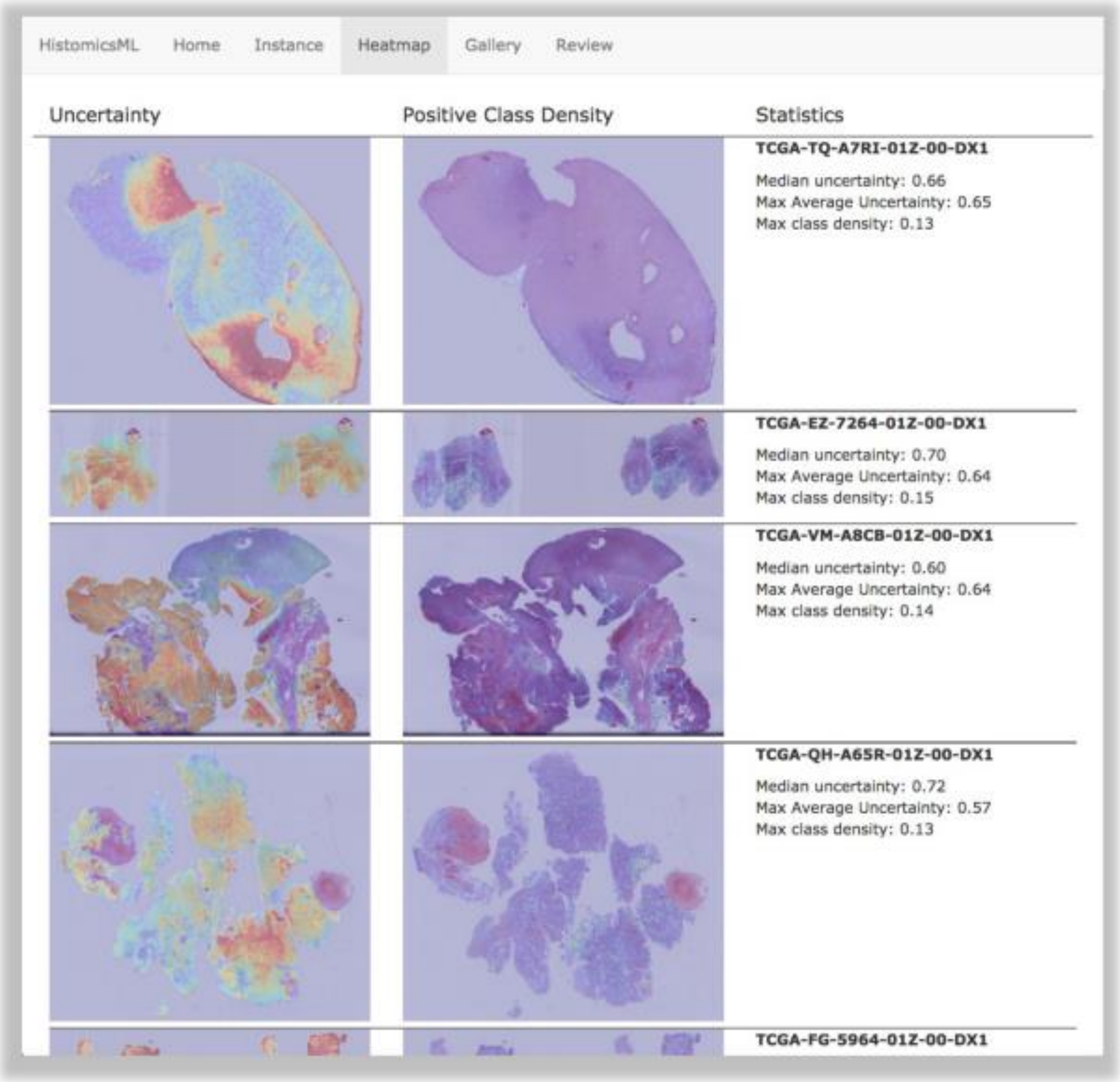

Figure S3E. Heatmap thumbnail gallery interface. This view displays slides overlaid with their confidence and positive class density heatmaps to prioritize feedback. Slides are sorted based on average confidence so that users can direct feedback to slides with large numbers of confounding samples. Clicking a thumbnail directs the user to the review screen for feedback. This page is updated and the slides resorted with each update of the classification rule.

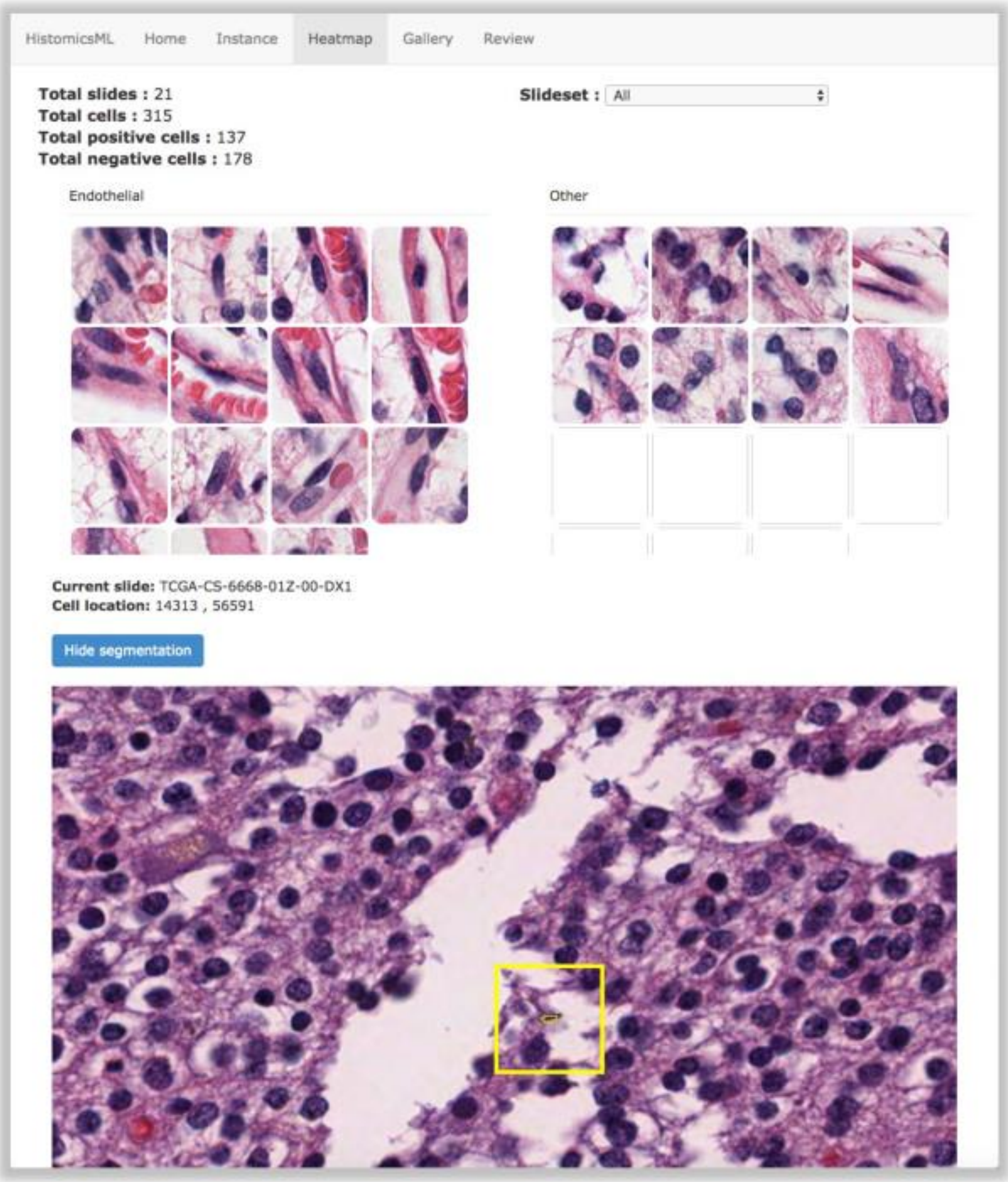

Figure S3F. Review interface. The review screen enables users to review and revise labeling provided for classification rule training. Labeled samples are organized in an array by slide and label/class. Users can browse the scrollable thumbnail gallery and change the label of a sample by drag-and-drop of the thumbnail images. Clicking a thumbnail directs the whole-slide viewport to the region of this sample.

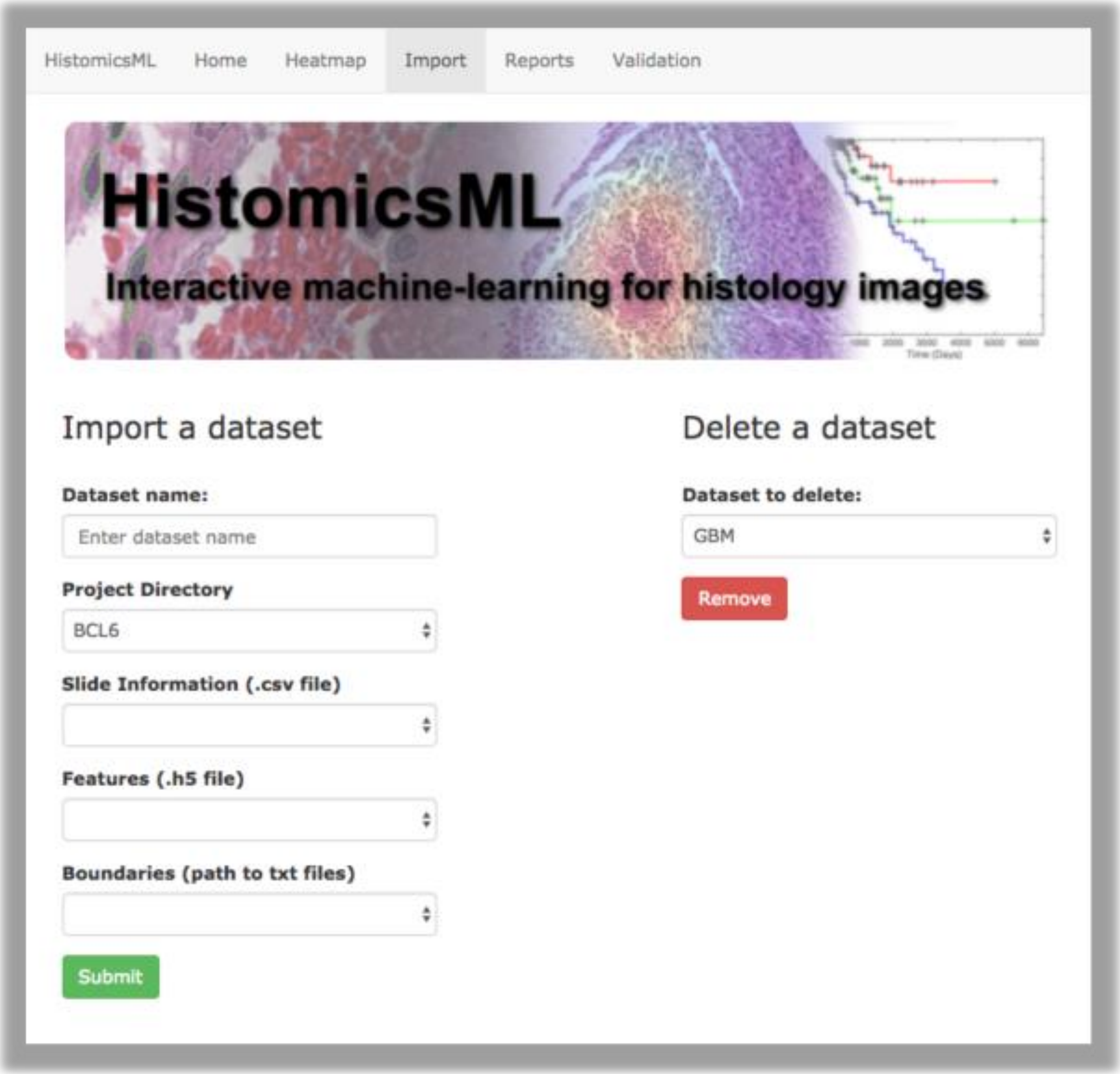

Figure S3G. Data import interface. The data import view can be used to import new datasets and to delete existing datasets. Data formats are described extensively in the documentation, and sample dataset files are provided in the Docker containers.

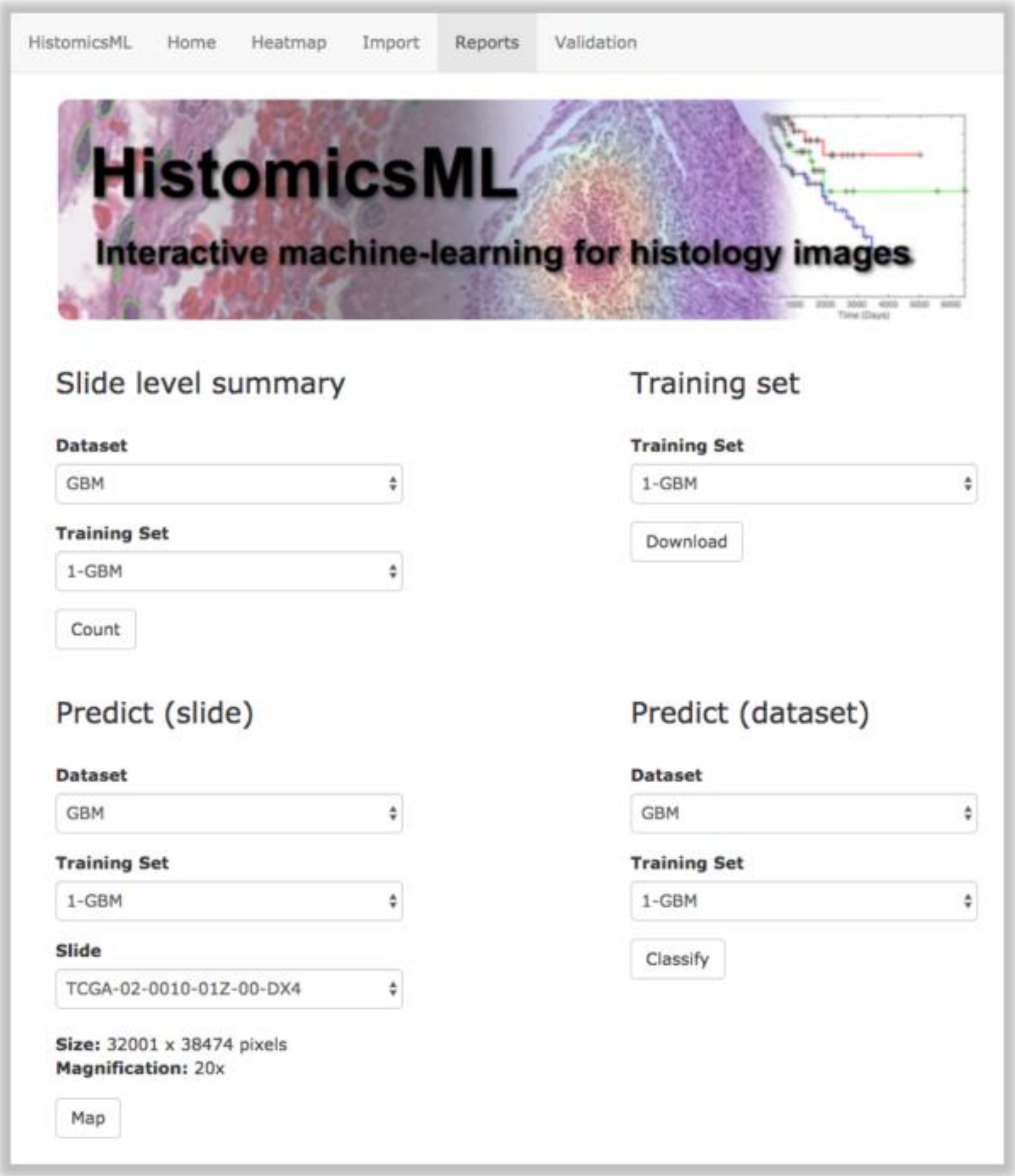

Figure S3H. Reports interface. The reports interface can be used to generate slide-level summaries of the classification results, deeper object-level summaries that capture the predicted class label, location (x,y), and prediction scores for individual nuclei in a dataset, and to download training sets for archival purposes.

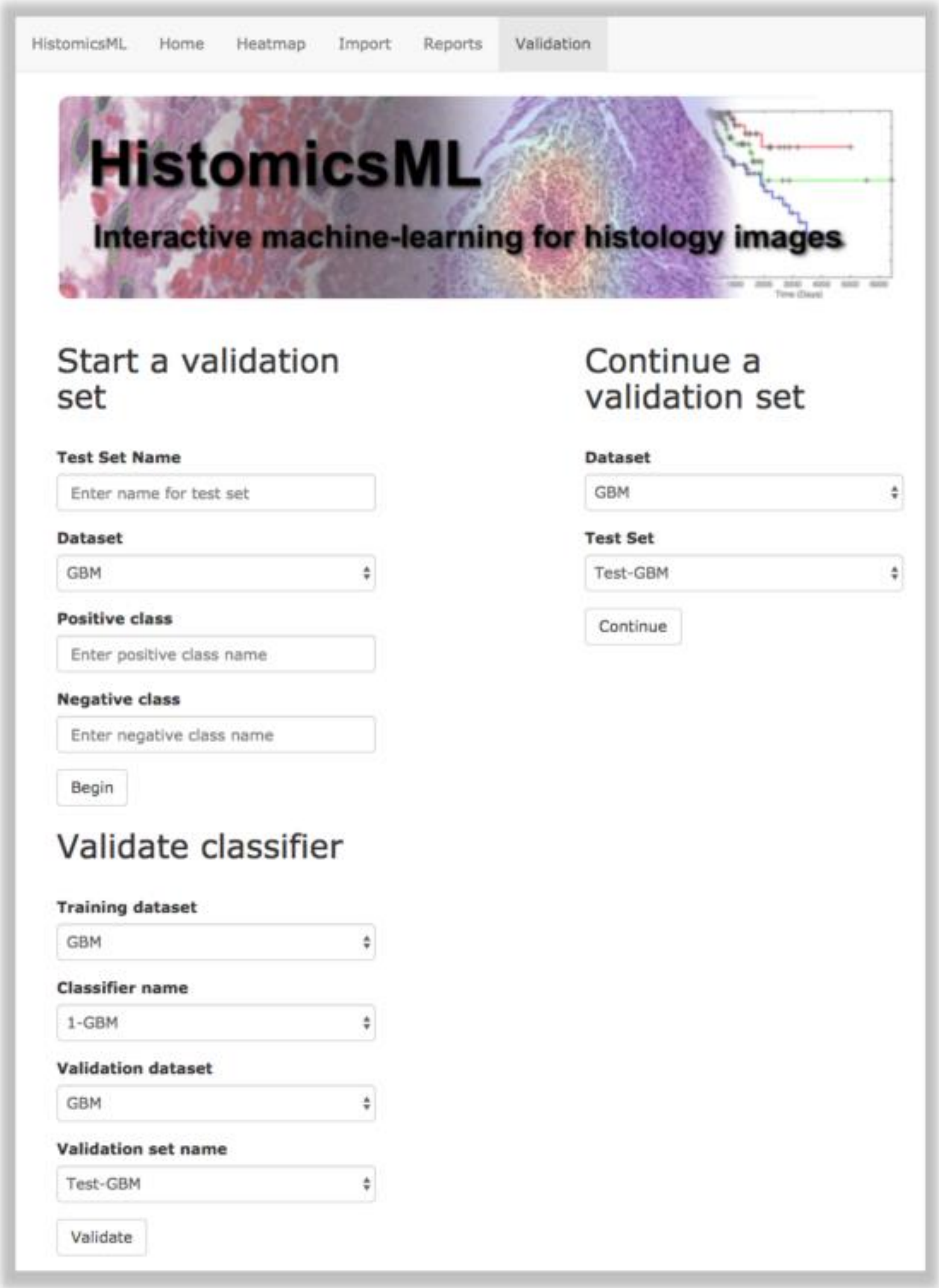

Figure S3I. Validation interface. The validation interface can be used to generate ground-truth validation datasets, providing a link to a whole slide viewer (as in Figures S3C, S3D) where objects can be annotated. These validation datasets can be used to validate the performance of classifiers, generating outputs that can be used to calculate statistics like accuracy, receiver-operating characteristic curves, sensitivity and specificity.

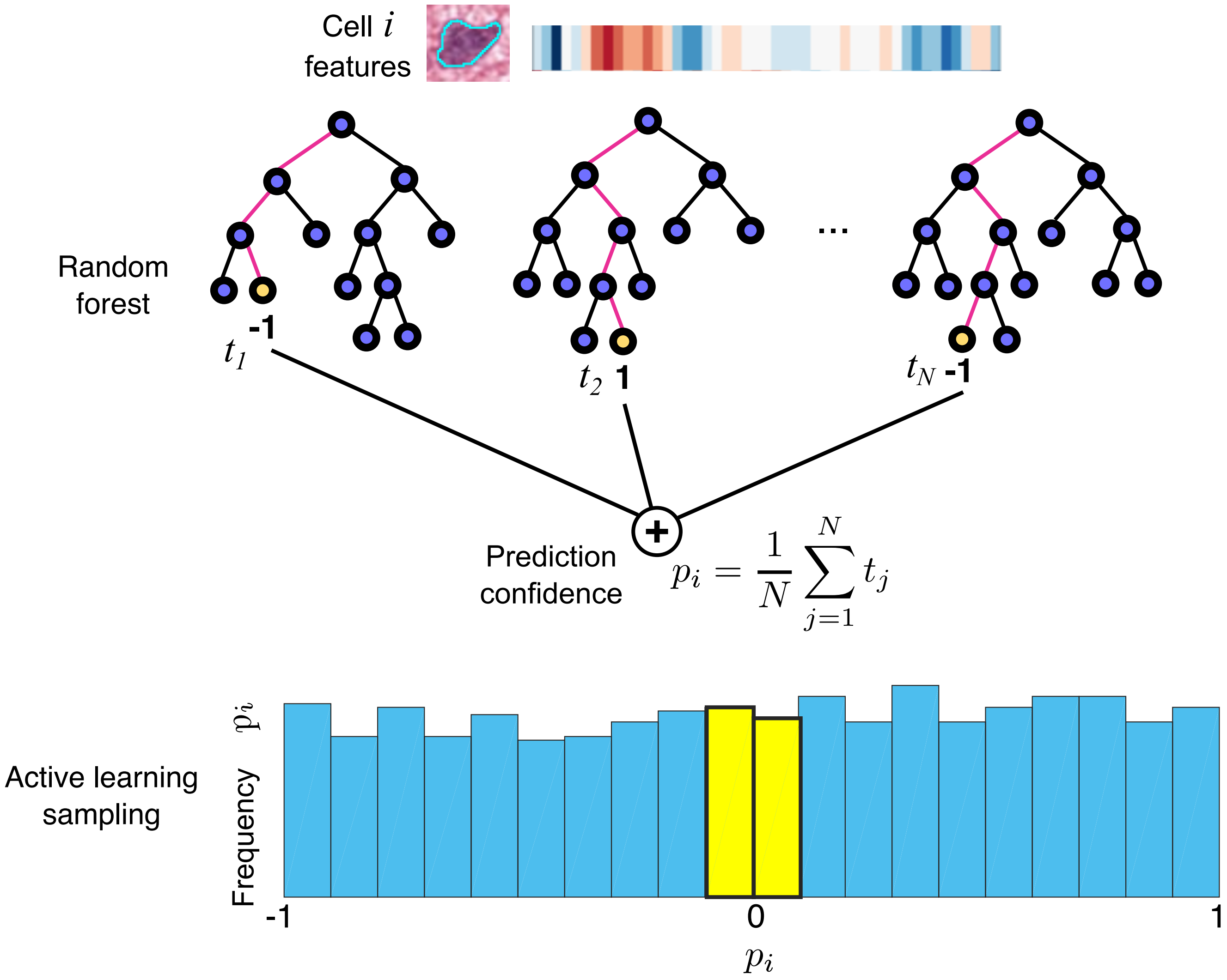

Figure S4. Active learning with random forests. The random forest classifier aggregates the predictions of multiple decision trees and provides a readout of prediction confidence. Given the histomic feature profile of an entity, each tree in the forest predicts the class t_j as either the positive (+1) or negative (-1) with the final aggregate prediction made by majority vote. Prediction confidence is measured as the absolute value of the prediction average (p_i). Objects with a confidence $| p_i |$ close to one indicate a consensus of the decision trees, where objects with a confidence $| p_i |$ close to zero indicate a lack of consensus by the trees. Objects with lower confidence scores are difficult to classify and make good candidates for labeling in the active learning paradigm. In our framework we calculate the object labels and confidence scores for instance-based sampling and heatmap generation with each classifier update/iteration. Objects with minimum confidence (where trees are tied or most discordant) are sampled for instance based learning.

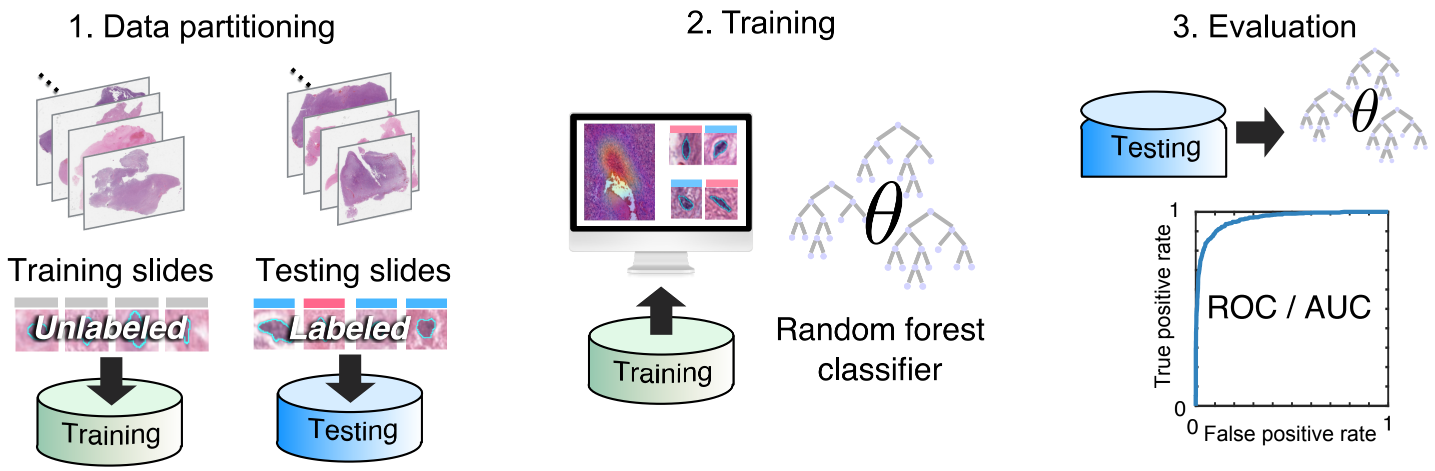

Figure S5. Validation of classifier performance. Our classifier of vascular endothelial cells was validated using independent sets of training and testing slides. Cell nuclei from testing slides were used with our system to generate a validation set of neuropathologist ground-truth labels. Cell nuclei from training slides were used to develop a classification rule for vascular endothelial cells using a combination of instance-based and heatmap facilitated learning. The accuracy of this classifier was evaluated on the labeled nuclei from the independent testing slides using receiver operating characteristic area-under-curve analysis.

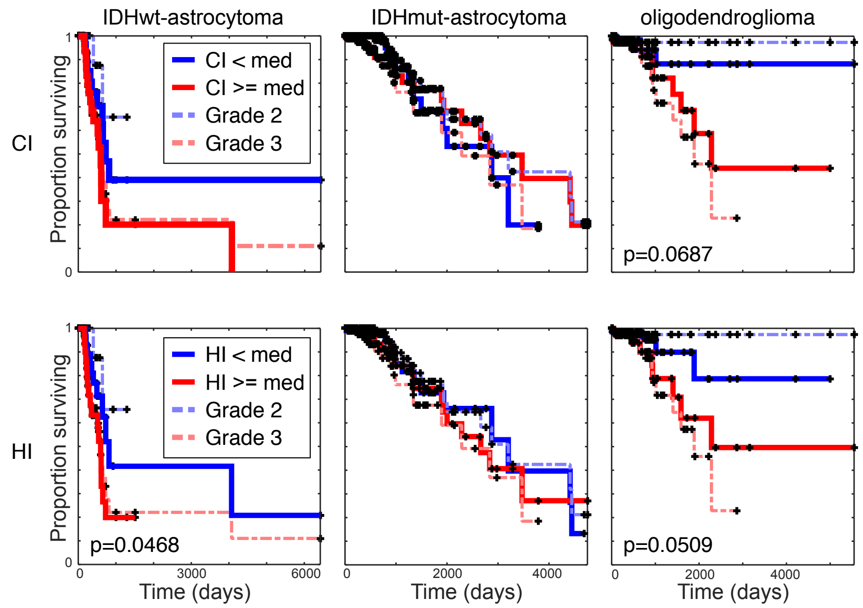

Figure S6. Kaplan-Meier analysis. Median values of CI or HI were used to stratify patients into low/high risk groups for Kaplan-Meier analysis in each molecular subtype (grade is shown for comparison).
